# Managing portal hypertension in patients with liver cirrhosis

**DOI:** 10.12688/f1000research.13943.1

**Published:** 2018-05-02

**Authors:** Tilman Sauerbruch, Robert Schierwagen, Jonel Trebicka

**Affiliations:** 1 Department of Internal Medicine, University of Bonn, Bonn, Germany; 2 European Foundation for Study of Chronic Liver Failure, Barcelona, Spain

**Keywords:** portal hypertension, liver cirrhosis, chronic liver disease

## Abstract

Portal hypertension is one cause and a part of a dynamic process triggered by chronic liver disease, mostly induced by alcohol or incorrect nutrition and less often by viral infections and autoimmune or genetic disease. Adequate staging - continuously modified by current knowledge - should guide the prevention and treatment of portal hypertension with defined endpoints. The main goals are interruption of etiology and prevention of complications followed, if necessary, by treatment of these. For the past few decades, shunts, mostly as intrahepatic stent bypass between portal and hepatic vein branches, have played an important role in the prevention of recurrent bleeding and ascites formation, although their impact on survival remains ambiguous. Systemic drugs, such as non-selective beta-blockers, statins, or antibiotics, reduce portal hypertension by decreasing intrahepatic resistance or portal tributary blood flow or by blunting inflammatory stimuli inside and outside the liver. Here, the interactions among the gut, liver, and brain are increasingly examined for new therapeutic options. There is no general panacea. The interruption of initiating factors is key. If not possible or if not possible in a timely manner, combined approaches should receive more attention before considering liver transplantation.

## Introduction

**Figure 1.**
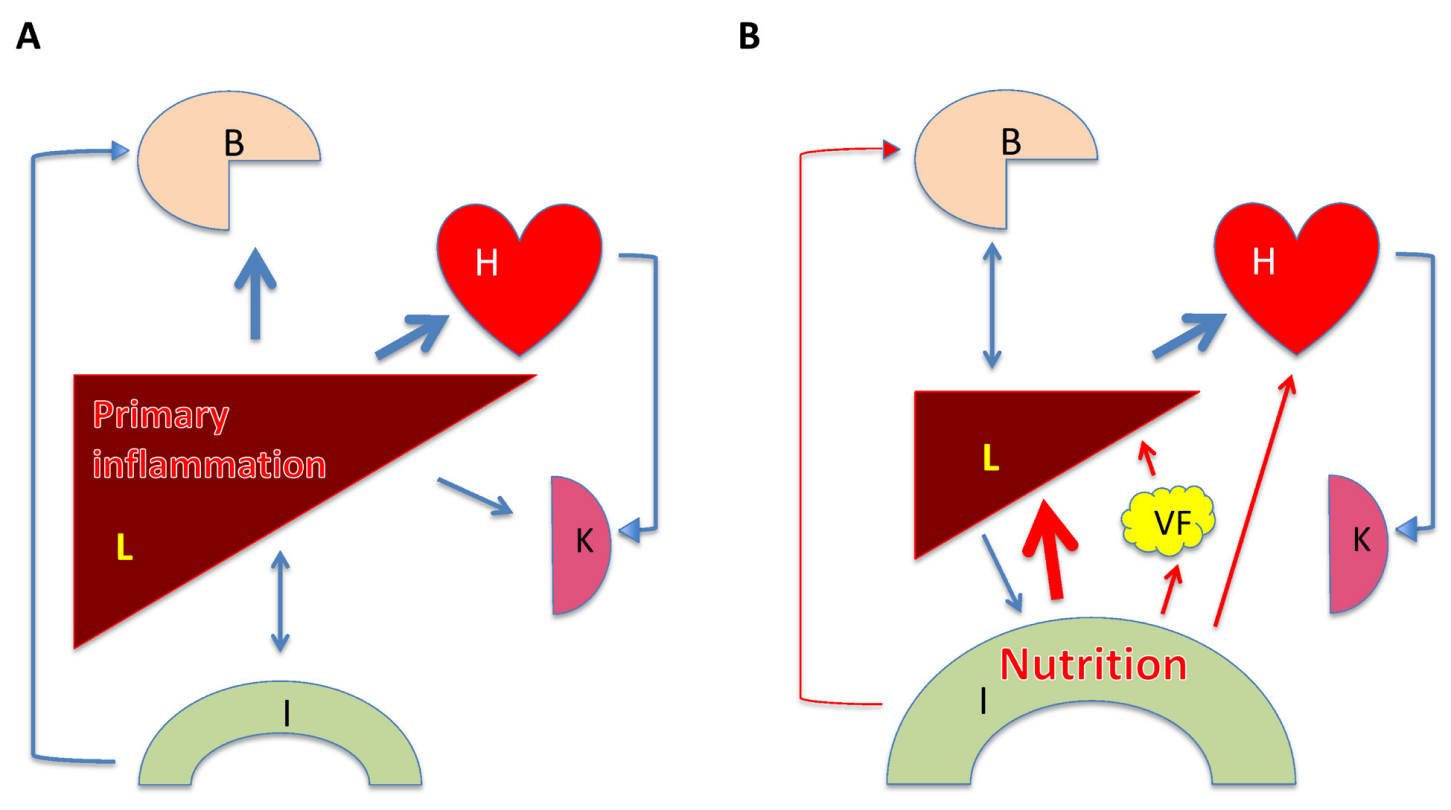
Two main pathways in the development of liver disease. **A**. The liver (L) is primarily affected (mainly by chronic infection with hepatotropic viruses). This leads to liver cirrhosis and portal hypertension causing leakiness of the intestine (I) and dysbiosis, affecting the brain ( **B**), and altering the splanchnic and systemic circulation, including the heart (H) and the kidneys (K). Prevention and therapy is interruption and/or suppression of viremia. **B**. Increasingly, nutrition has become the main culprit in liver disease. Here, inflammatory and metabolic stimuli from the gut affect the liver, visceral fat (VF), and cardiovascular system, including the heart and the kidneys, but also, concomitantly and early on in the process, the brain, which may support a vicious cycle of craving more food and liquids. Prevention and therapy is modification of food and liquid intake. Only in the later stages of liver disease do the complications of liver cirrhosis and portal hypertension determine pathogenesis, diagnosis, and therapy similar to **A**).

**Figure 2.**
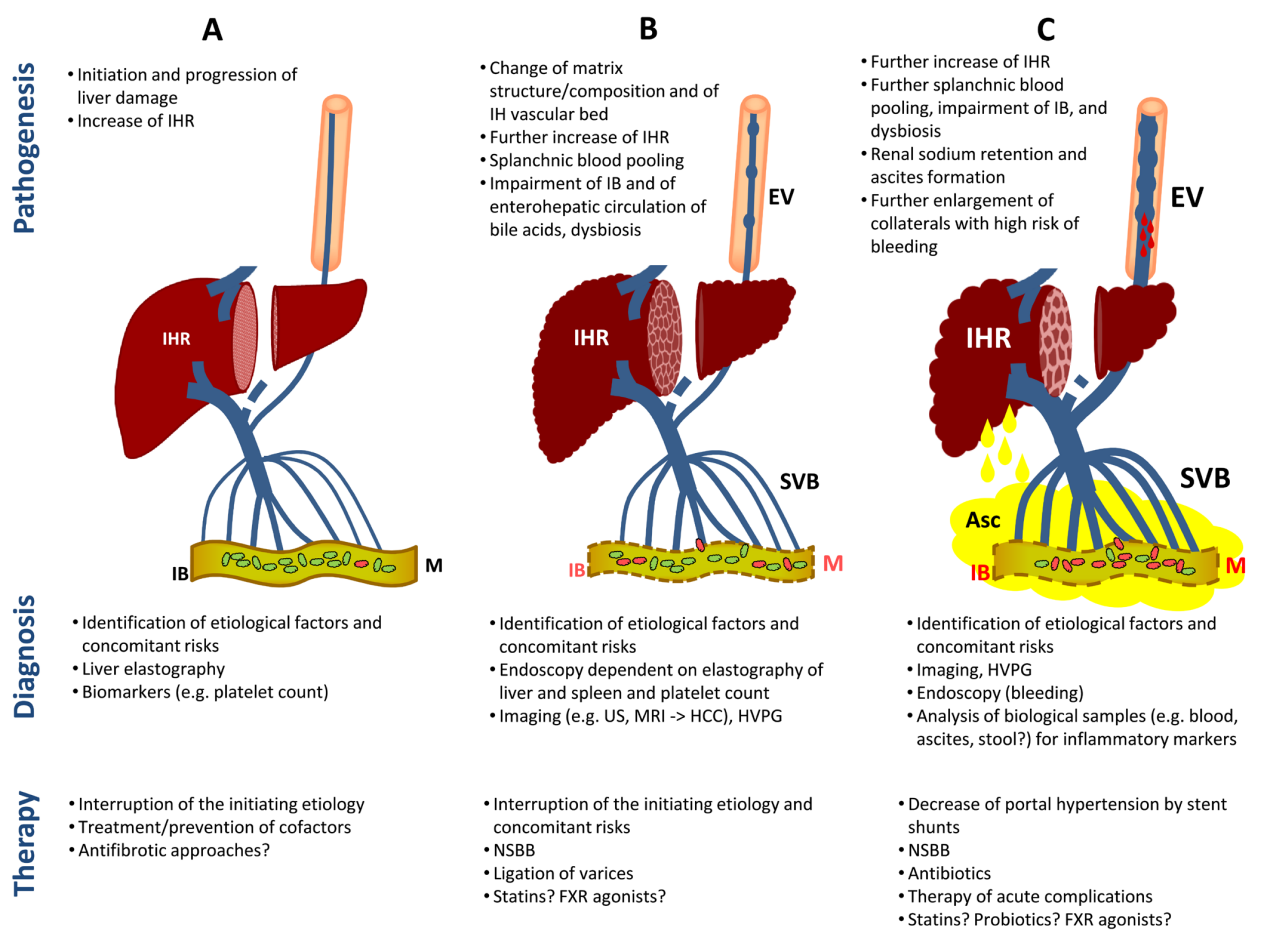
Stages of chronic liver disease and portal hypertension. The figure depicts the diagnostic and therapeutic procedures during the pathogenesis and aggravation of portal hypertension for patients with suspected fibrosis/cirrhosis of the liver ( **A**), compensated cirrhosis ( **B**), and decompensated cirrhosis ( **C**). Asc, ascites; EV, esophageal varices; FXR, farnesoid X receptor; HCC, hepatocellular carcinoma; HVPG, hepatic venous pressure gradient; IB, intestinal barrier; IH, intrahepatic; IHR, intrahepatic resistance; M, microbiome; MRI, magnet resonance imaging; NSBB, non-selective beta-blocker; SVB, splanchnic vascular bed; US, ultrasound.

Portal hypertension is defined as the pathological increase of portal venous pressure, mainly due to chronic end-stage liver disease, leading to augmented hepatic vascular resistance and congestion of the blood in the portal venous system. This pathology may result in a series of complications, such as the formation of collateral vessels for return of the blood to the right atrium with potential for intestinal bleeding, formation of ascites, encephalopathy, and development of a hyperdynamic circulation involving peripheral and splanchnic vessels ^1, 2^ associated with dysfunction of the kidneys ^3^, the heart ^4^, the lungs ^5^, and the brain ^6^ ( Figure 1 and Figure 2). A complex interplay among inflammatory stimuli, vasoregulatory molecules, neurotransmitters, and ion channels maintains and drives these processes. Thus, portal hypertension is one cause and a part of a dynamic process triggered by chronic liver disease and systemic inflammation ^7^. In the stage of advanced liver disease, mostly fixed structural changes, such as fibrosis or the formation of regenerative nodules, are responsible for developing and sustaining portal hypertension. In addition, dynamic components involving the regulation of blood flow in different vascular beds play a decisive role in the modulation of portal pressure and its associated pathophysiology. Systemic therapy is aimed at the modulation of these dynamic parts. Most likely, they are more or less similar in end-stage liver disease, regardless of the etiology of hepatic damage. However, in the early stages of liver disease, the pathological chain of events depends more on the causative factors, be they metabolic, infectious, or autoimmune. Thus, early and specific treatment is the foremost aim. During later stages of liver disease, distinct treatment of the initiating factors may still be pivotal, i.e. interruption of viremia or alcohol abuse ( Figure 1 and Figure 2). However, this must be combined with measures to prevent or treat complications due to liver cirrhosis.

This review focuses on portal hypertension in patients with advanced liver disease. It will address some open questions and new approaches on how to stage chronic liver disease and portal hypertension and how to prevent some of its complications.

## Staging of chronic liver disease and portal hypertension

For staging of chronic liver disease, a variety of different tools are available, including physical examination, laboratory tests, imaging techniques, and hemodynamic measurements ( Figure 2). Imaging techniques comprise endoscopy, ultrasound, determination of liver stiffness, computed tomography, and magnetic resonance imaging (MRI). Physical examination includes important parameters of the Child–Pugh classification ^8^. If there are no signs of jaundice, ascites, or encephalopathy, the patient has a good chance of being in a compensated stage of cirrhosis with a 10-year survival of above 50%, while clinical signs of decompensation indicate a mortality of more than 75% within the next 5 years ^9^.

Endoscopy is still the best method to assess the existence of varices in the upper intestinal tract as well as their size and potential to bleed or rebleed ^10^. While in any patient with suspected liver cirrhosis a standard examination used to include endoscopy, new guidelines recommend abstaining from early endoscopy in patients with liver stiffness <20 kPa and platelet count >150 G/L ^11, 12^. These patients have a high probability of being free of esophageal varices ^13^. However, endoscopy retains its central role as the entrance test for the initiation of primary and secondary prophylaxis of variceal bleeding in patients with higher stiffness values or a lower platelet count, and it is still the central method for the assessment of variceal bleeding and hemostasis ^12^.

In addition, elastographic techniques enable estimation of the degree of liver fibrosis via transient elastography (TE), acoustic radiation force impulse imaging (ARFI), or shear wave elastography (SWE) ^14, 15^. Determination of liver stiffness has by now become an important tool for screening of fibrosis and portal hypertension in patients with liver disease. Fibrosis leads to an increased stiffness of the liver. In organs with higher stiffness, shear waves travel with a higher speed through tissues. By delivering pulses, shear waves can be induced to assess their speed as an indirect measure of fibrosis. There are different systems using mechanical 50 Hz pulses (TE), a focused ultrasound pulse to deform internal tissue (AFRI and SWE), or a two-dimensional gradient-recalled-echo sequence analyzed by certain algorithms (magnetic resonance [MR] elastography).

The most extensive experience to date exists for TE, a stand-alone technique based on shear wave speed measurement, not integrated into ultrasound devices ^16– 18^. Values below 5.2–9.5 kPa (TE) or 1.22–1.63 m/s (ARFI) can rule out significant liver fibrosis, whereas higher values may be falsely positive with respect to cirrhosis assessment because of obstructive cholestasis, liver congestion, severe liver inflammation, or infiltrative liver disease ^16, 17^. However, many of these obscuring conditions can be assessed or ruled out by using ultrasound-based techniques such as SWE or AFRI. SWE has shown slightly better sensitivity and specificity for liver fibrosis and portal hypertension when compared to TE ^19^. Nevertheless, observing standardized conditions like fasting state is important ^20^. By combining liver and spleen SWE, portal hypertension can be excluded with a very high probability ^21^ on the one hand or assessed in its clinically significant form on the other ^22^. Furthermore, ultrasound-based techniques allow for hepatocellular carcinoma (HCC) screening.

The different systems have pros and cons. TE is available in many centers and is excellently validated but may have a high failure rate in obese patients or in patients with ascites. AFRI allows ultrasound guidance for the region of interest but is less validated, and high body weight may also be a problem. MR elastography allows one to cover a large sampling volume, but it is affected by iron deposition, high body mass index, and massive ascites ^23^.

Ultrasound allows a more sensitive and specific assessment of ascites than clinical examination together with assessment of size, surface, and echotexture of the liver.

Similar to ultrasound, computed tomography and MRI are relevant for the diagnosis of HCC. This is important, since liver fibrosis or cirrhosis is a precancerous condition. Of all imaging devices, MRI has the broadest potential for staging liver disease with respect to morphology, including the biliary system, tissue texture, perfusion, formation of collaterals, function of hepatic cells ^24– 26^, and quantification of steatosis or fibrosis ^27, 28^. However, it is expensive and not always available. We recently showed how MRI can be used to measure fat-free muscle area as a prognostic marker of sarcopenia, correlated with survival ^29^ in patients with liver cirrhosis.

Among hemodynamic measurements, assessment of the hepatic venous pressure gradient (HVPG) ^30^ is the most important in chronic liver disease. Developed in the 1950s ^31^ and later modified by Groszmann *et al*. ^32^, it has become the gold standard for indirect assessment of the degree of portal hypertension. The HVPG value closely correlates with the portal vein pressure, especially in alcoholic liver disease ^33^, which in turn shows a significant correlation with blood pressure in esophageal varices ^34^. HVPG values above 5 mmHg are regarded as portal hypertension. Measurement of the HVPG adds prognostic information to standard laboratory and clinical evaluations in advanced liver disease ^35, 36^. Patients with compensated cirrhosis and HVPG <10 mmHg have a rather low risk of developing varices or decompensation of liver function ^37^. It is generally accepted that esophageal varices do not bleed if HVPG remains below 12 mmHg and that a reduction of HVPG by more than 20%, regardless of the baseline value, considerably reduces the risk of bleeding from varices. Thus, measurement of HVPG has repeatedly been advocated as a means to tailor the treatment for variceal bleeding ^35, 38^. There is a good correlation between liver stiffness, as assessed by TE, and HVPG ^39^. Values below 14 kPa exclude clinically significant portal hypertension (HVPG ≥10 mmHg) with high sensitivity and specificity ^40^.

Since the introduction of the Child–Turcotte classification ^8^ and its modification according to Pugh *et al*. ^41^, it has been repeatedly shown that, in patients with liver cirrhosis, laboratory values reflecting hepatocyte function, e.g. uptake and secretion of bilirubin or synthesis of proteins, allow prediction of the probability of survival. Thus, serum levels of bilirubin, albumin, or clotting factors have been used for decades to stage chronic liver disease. They are part of the model for end-stage liver disease (MELD) system ^42^ as well as of the Child–Pugh classification ^41^.

The MELD consists of serum levels of bilirubin and creatinine and prothrombin time determined as an international normalized ratio (INR). Initially developed to determine the prognosis of patients receiving a transjugular intrahepatic portosystemic shunt (TIPS) ^42, 43^, it is now used to assess organ allocation priority for liver transplantation. It can be calculated easily, has been validated prospectively in different cohorts, and contains no clinical parameters based on subjective assessment. Nevertheless, MELD is only slightly superior to the Child–Pugh model in the prediction of survival ^44, 45^. The addition of further parameters such as sodium ^46^, hepatic encephalopathy ^45^, or sarcopenia ^47^ to MELD has been described to further improve prognosis with marginal effects.

Impairment of kidney function, such as sodium handling, occurs early in patients with liver disease ^48^, and elevated creatinine levels—or, more importantly, an increase in serum creatinine by ≥0.3 mg/dL - are independent markers for negative patient outcome ^49^.

Patients with liver cirrhosis are prone to systemic inflammation, where mostly cytokines of the innate immune system are involved ^50^. This paves the way towards fatal dysfunction of organs, now coined acute-on-chronic liver failure (ACLF) ^51^. Thus, signs of inflammation, such as high leukocyte count and elevated C-reactive protein, are additional important prognostic parameters ^52^. Interestingly, there is a subgroup of patients who, even after TIPS insertion, show an increased or unchanged liver stiffness. These are patients with high levels of proinflammatory cytokines. They have a bad outcome ^53^. Thus, dynamics in liver stiffness may be an easy read-out to assess inflammation and prognosis in TIPS patients. Furthermore, the individual genetic background with respect to genes coding for proteins involved in the immune response may predispose patients to infections, ACLF, and decompensation ^54– 58^.

Many of the above-mentioned parameters are part of staging systems for liver cirrhosis designed to distinguish between compensated and decompensated disease at different states ^59^. If it is true that the intestine is important for the initiation and perpetuation of liver disease (see below), we will also need some sort of staging for intestinal dysfunction in patients with liver disease in the future.

The different staging systems mentioned above, such as the degree of fibrosis, HVPG, ongoing etiology, Child–Pugh, dynamics of kidney dysfunction, or signs of inflammation, are partly interrelated. Thus, HVPG increases with the degree of cirrhosis ^60, 61^ or the degree of decompensation as assessed by the Child–Pugh score. However, the correlation is loose, and the prognostic value of HVPG is partly independent of the Child–Pugh system ^62, 63^. Therefore, there is always the question of how to integrate different parameters or scores into an appropriate and simple bedside system. Clinical judgment is quite accurate for advanced liver disease. Jaundice and ascites are markers of bad prognosis. In this situation, bleeding, infections, overt encephalopathy, and deterioration of kidney function denote high risk of death. Determination of HVPG and/or of liver stiffness may improve long-term prognosis in patients with compensated cirrhosis ^59^, and HVPG alone is an independent prognostic marker in patients with decompensated cirrhosis and variceal bleeding ^36, 62, 63^. In the future, we will probably have to adapt to more complex systems ^59, 64^ which might improve prognosis and therapy. No matter which stage of disease, the interruption of etiology, be it alcohol intake or viremia, is crucial.

As with all staging systems in medicine, the question arises as to whether these surrogates or biomarkers can guide the prevention or treatment of relevant clinical endpoints in patients with portal hypertension. The following sections will address some issues in this puzzle.

## Decrease of portal pressure by shunt procedures

The most effective measure to reduce portal hypertension is to circumvent the increased intrahepatic resistance in liver cirrhosis and bypass the blood into the inferior vena cava by portacaval, mesocaval, or proximal splenorenal shunts. Controlled trials evaluating the potential of a surgical open shunt procedure were mainly performed to assess their effect on the prevention of bleeding from varices. They date back to the 1960s ^65– 67^, but the most current long-term follow-up studies have been published as recently as 2012 and 2014 ^68, 69^. The indication for open shunt procedures was almost exclusively prevention of bleeding. Although open surgical shunts may have advantages in young patients with severe portal hypertension, recurrent bleeding, and good liver function, this surgical procedure has been more or less abandoned and surgical experience is waning. This is mainly owing to its invasiveness, perioperative mortality, and an increased risk of liver failure and/or encephalopathy due to loss of liver perfusion with portal venous blood. Accordingly, it has never been convincingly shown that surgical shunts improve survival. By contrast, transjugular insertion of an intrahepatic stent between a branch of the portal vein and a branch of the hepatic vein (TIPS) is less invasive and has become an established treatment approach in portal hypertension and its complications. After a learning period in different pioneering centers ^70^, the procedure is now established worldwide. In most patients, TIPS implantation reduces portal pressure by more than 50%, as assessed by the portal pressure gradient. The degree of reduction depends on the diameter of the stent ^36, 70^. TIPS prevents variceal rebleeding in the vast majority of patients. According to many controlled trials and respective meta-analyses, TIPS is superior to ligation of varices with or without the addition of beta-blockers ^71^. Yet the combination of ligation and beta-blockers (see below) is still considered the procedure of choice for rebleeding prophylaxis ^11^, mainly because TIPS patients with decompensated cirrhosis (bilirubin >3–5 mg/dL) are suboptimal candidates for shunt insertion, as they have a relatively high risk of liver and mental function deterioration. In the elective situation, TIPS implantation is therefore mainly used as a potential rescue procedure for the treatment of rebleeding of esophageal varices or the treatment of refractory ascites. According to randomized trials, around 20% of patients receiving local endoscopic rebleeding prophylaxis have to be switched to TIPS implantation because of refractory ascites or recurrent bleeding events ^36, 72– 74^. Thus, in patients with variceal bleeding and ascites, early placement of a small lumen-covered TIPS should be earnestly considered as early therapy.

One disadvantage of TIPS is shunt occlusions of bare stents, a rare event after shunt operation ^75^. However, this problem has been solved to a large extent by the introduction of polytetrafluoroethylene-covered stents ^76, 77^. Furthermore, there is evidence that the placement of small-diameter, covered stents (8 mm) reduces the encephalopathy rate ^36, 78^, while its protection from rebleeding remains. But, unfortunately, even small covered stents are still burdened with the risk of encephalopathy ^36^. Although TIPS insertion is now the most efficient method to reduce portal hypertension and to prevent bleeding in patients with liver cirrhosis, it does not improve survival as compared to patients receiving a non-shunt approach ^71^, at least in the elective situation. This also holds true for the most recent trials comparing non-selective beta-blockers (NSBB) with or without ligation to TIPS with covered stents ^36, 73, 79^.

Trials suggest that pre-emptive or “early” TIPS insertion is beneficial in high-risk patients, mainly those with active bleeding, decompensated liver cirrhosis, and/or HVPG >20 mmHg ^62, 80, 81^, with respect to not only hemostasis and early rebleeding but also long-term survival. However, this strategy still needs to be established and proven in broad clinical practice. Currently, early TIPS insertion for acute variceal hemorrhage is neither always available nor widely applied in the real-world scenario ^82^. The positive effect of TIPS insertion for the prevention of bleeding in patients with liver cirrhosis declines with increasing temporal distance to the index bleeding event in the acute ^83^ and elective ^36^ situation, i.e. for the treatment of bleeding in patients with liver cirrhosis, suitable patients together with the appropriate time window ^84^ have to be identified.

TIPS has a positive impact on hemodynamic changes in liver cirrhosis. Central blood volume and cardiac output increase after shunt placement ^85, 86^. This is associated with a deactivation of the renin–angiotensin–aldosterone system (RAAS) and improvement of impaired kidney sodium excretion in liver cirrhosis ^70, 85, 87^. It explains the positive effect of TIPS insertion for the mobilization of refractory ascites. While this method was initially developed to prevent intestinal hemorrhage in liver cirrhosis, the number of patients receiving stents for the treatment of ascites now surpasses the bleeding indication according to our own experience ^53^ (unpublished data of groups from Bonn/Freiburg). There is an ongoing debate on the role of TIPS, especially with respect to the survival of ascitic patients, in comparison to paracentesis with albumin infusion ^88^. Analysis of the early studies using bare stents already suggested that TIPS improves transplant-free survival in patients with refractory ascites ^89^. A recent randomized study with limited patient numbers using covered stents showed a highly significant improvement of transplant-free survival in patients who had received covered TIPS for recurrent ascites when compared to paracentesis with albumin ^90^.

Taken together, TIPS, especially with technically improved stents, has become well established in the prevention and treatment of intestinal bleeding and ascites in patients with liver cirrhosis. However, the selection of patients is key. By contrast, hardly any centers exist that still perform shunt surgery for portal hypertension.

Shunts, including TIPS, bypass the increased hepatic resistance in patients with liver cirrhosis and exert their beneficial effect by a shift of the blood pool from the splanchnic to the central venous compartment. Most drugs, by contrast, act more by blunting stimuli that are activated or overactivated in liver cirrhosis. Some of these are addressed in the following paragraphs.

## Modification of portal pressure by non-specific drugs

New and old pathogenetic concepts showed that advanced liver cirrhosis with portal hypertension is a systemic disease involving most organs. It remains a challenge to counter this. The interruption of etiology is the most important step, mainly with respect to the progression of liver disease but also with respect to an immediate portal pressure-lowering effect. This holds true for the interruption of hepatitis C ^91– 93^ or abstinence from alcohol ^94^. Since chronic alcoholism is now the most frequent cause of liver cirrhosis and portal hypertension in most countries, we need a more holistic approach to alcohol use disorders ^95, 96^.

In the following sections, we refer to medical treatments that are not curative but may have beneficial adjuvant effects.

### Non-selective beta-blockers (NSBB)

The concept of treating portal hypertension with NSBB was introduced nearly four decades ago ^1^ by a French group under the hypothesis that the portal tributary blood flow is increased in liver cirrhosis with portal hypertension and that NSBB decrease portal flow and pressure by reducing the cardiac index and splanchnic vasodilatation. The concept proved to be right, but the achieved reduction in portal pressure is only about 15% on average ^36, 97^. Numerous randomized controlled studies have documented the privileged place of NSBB in the treatment of portal hypertension, mainly for the prevention of first bleeding and, combined with endoscopic ligation, for recurrent bleeding ^11, 98^. However, for the prevention of first bleeding, sole ligation of esophageal varices is at least equivalent ^99^ if not better ^100^ than NSBB, especially in patients with large varices ^11^. The combination of NSBB and ligation has no advantage in this setting. Although it has been known since the mid-1990s ^101^ that only patients with adequate pressure reduction (>20% or to <12 mmHg), as assessed by HVPG, are sufficiently protected from rebleeding, no suitable controlled studies have been published that address the question of whether the application of NSBB tailored by hemodynamic control (HVPG measurement) is superior to the application of NSBB in any patient for primary bleeding prophylaxis. Unfortunately, sufficient (>20%) portal pressure reduction is achieved in only around 40% of patients ^36, 102^, and almost one-third of patients with liver cirrhosis have contraindications to NSBB, experience side effects, or are non-compliant ^99^.

A recent study on rebleeding prevention suggests that patients with liver cirrhosis who show a hemodynamic response to NSBB have an improved survival compared to those who fail to respond ^103^. However, the debate remains controversial as to whether or not the continuation of NSBB may even worsen the outcome in non-responders ^104, 105^. This has never been systematically evaluated. It is worth noting that it has been argued in this context that NSBB, besides their effect on splanchnic hemodynamics, modulate systemic inflammation in liver cirrhosis ^106^. This might explain why patients with cirrhosis and ACLF who had received NSBB within 3 months before admission (half of them were kept on NSBB also after admission) fared somewhat better than those without NSBB, but long-term survival was not different ^107^.

A French publication ^108^ initiated the debate of whether NSBB should be omitted in patients with refractory ascites, in whom they may cause deleterious hemodynamic dysfunction, or in patients after infection of ascites ^109^. To date, most experts agree that only a systolic blood pressure below 90 mmHg and signs of worsening kidney function are caveats for continuation of NSBB ^11^, at least in higher dosages ^110^.

The type of NSBB for patients with liver cirrhosis has become an issue since it has been shown that, in patients with cirrhosis, carvedilol, a NSBB with additional alpha-1 adrenoceptor-blocking properties, induces a better hemodynamic response ^97, 111^, as determined by HVPG drop, than propranolol or nadolol and prevents the progression of small esophageal varices ^112^, an effect not found with propranolol. Retrospective data even asserted the prolongation of survival in patients with cirrhosis and ascites receiving carvedilol ^113^, while a recent letter analyzing several clinical studies drew the conclusion that carvedilol may even increase mortality compared to propranolol and nadolol ^114^. All this has to be considered with caution until sufficient randomized trials with predefined endpoints have been performed. Current data are too limited ^115^. Regardless of the NSBB type used, particular attention must be given to the hemodynamic status of a patient with liver cirrhosis, especially in the case of concomitant severe ascites, kidney dysfunction, reduced cardiac output, and/or infection.

In summary, NSBB kept their place for decades in the management of portal hypertension, mainly for the prevention of first or recurrent bleeding from varices. They may have an additional pleiotropic effect on reducing infectious stimuli from the gut. Caution is required in patients with severely decompensated cirrhosis or hemodynamic instability.

### Statins: a potential therapy for portal hypertension?

NSBB primarily target the dysfunctional cardiovascular system outside the diseased liver. In the last two decades, clinical research has concentrated on the paradox that patients with liver cirrhosis exhibit splanchnic and systemic vasodilatation while their blood perfusion through the liver is hampered by an increased and unopposed activation of intrahepatic contractile cells, apart from structural changes due to fibrosis, capillarization of sinusoids, or regenerative nodules ^116^. A crucial step in this process is the activation and transdifferentiation of hepatic stellate cells in the space of Disse together with a dysfunction of sinusoidal cells ^117^ caused by numerous different stimuli reaching the liver. Many more recent strategies for the treatment of portal hypertension aim to modulate this chronic intrahepatic hyper-responsive inflammatory process and its defects. Here, statins have been the focus for many years. There are numerous reports on the pleiotropic effects of these drugs apart from their LDL cholesterol-lowering benefit, some of which are relevant for liver disease ^118– 121^. Important among these are increased intrahepatic formation of the vasodilator nitric oxide ^122^, downregulation of signaling molecules that activate hepatic stellate cells ^122– 124^ by reduced prenylation of small GTPases, modulation of the crosstalk between hepatic stellate cells and endothelial cells, upregulation of transcription factors that restore intrahepatic endothelial function ^125^, and downregulation of intrahepatic inflammatory cytokines ^126, 127^. All of these effects explain the reduction of intrahepatic resistance with a drop in portal pressure ^122, 128, 129^ and blunting of collagen formation in experimental liver cirrhosis ^123, 126, 127^. A first randomized clinical trial on rebleeding prophylaxis in patients with liver cirrhosis who had succumbed to variceal hemorrhage could not confirm the hypothesis that the addition of statins to standard rebleeding prophylaxis (ligation and NSBB) further reduces the probability of rebleeding. However, compared to placebo, survival was better in the statin group ^130^. Mortality of the bleeding events and the infection rate were lower in patients receiving statins. This may be explained by the observation that statins have anti-inflammatory and immunomodulatory effects (for further details, see 118).

Large retrospective studies have shown that statins reduce the risk of cirrhosis and its decompensation in chronic hepatitis B- and hepatitis C-associated liver disease ^131– 134^, possibly because of their anti-inflammatory effect within the liver ^126, 127^. Thus, statins might primarily find their role as an adjuvant treatment to retard the progression of cirrhosis and portal hypertension with its complications in patients in whom a timely interruption of the etiology of chronic liver disease was not possible. In these patients, the potential hepatotoxicity of statins is a minor problem ^135, 136^. However, in patients with decompensated cirrhosis, particular attention must be paid to adverse events ^130^. Animal studies found that nitric oxide-donating statins may be as effective but less toxic ^137^.

## Modulation of the intestine

Intrahepatic resistance, portal tributary blood flow, and formation of spontaneous shunts determine portal pressure ^116, 138^. Intrahepatic resistance has a structural (fibrosis, alteration of the vascular architecture) and a non-structural (intrahepatic vascular tone) component. Gut-derived stimuli such as certain pathogen-associated molecular patterns (PAMPS) (lipopolysaccharide, endotoxin) may play an important role in this interplay. They impair intrahepatic endothelial function by influencing nitric oxide release ^138, 139^ or stimulate signaling pathways of intrahepatic contractile cells and of fibrogenesis by inducing inflammation, e.g. activation of Kupffer cells and other macrophages ^140, 141^ using sensing protein families such as Nod-like receptors or Toll-like receptors ^142, 143^. Especially in fatty liver disease, an early increase in portal pressure may be caused by functional non-structural changes of the intrahepatic vascular bed ^144^. Many questions in this context are not resolved. We will touch on some aspects in the following paragraphs.

Alcohol is the most common cause of progressive liver dysfunction and portal hypertension in the Western world ^145^, although only a minority of heavy drinkers develop liver cirrhosis ^146, 147^. Early research on the mechanism of alcohol-induced liver damage focused on direct or indirect hepatotoxic effects of ethanol and its oxidative and non-oxidative metabolites, such as acetaldehyde or fatty acid ethyl esters ^148^. The handling of fatty acids by hepatocytes was also studied, since hepatic steatosis is an important pre-stage of alcoholic liver cirrhosis. There is abundant literature on this, but it is mostly based on animal and cell culture models. Later, endotoxin from the outer membrane of Gram-negative bacteria was more and more regarded as a cofactor for the pathogenesis of alcoholic liver disease ^148, 149^ as a gut-derived stimulus. Further research showed that it mediates intrahepatic inflammation via Toll-like receptor 4 on macrophages ^150^, which in turn are a trigger for portal hypertension ^140^. Thus, the intestine has become a prime target for research on alcoholic liver disease and the catchword of today is gut–liver axis.

Our intestine, mainly the colon, is host to an enormous number of microorganisms, such as bacteria, fungi, Archaea, and viruses ^151^. Most of these microorganisms are commensals with a symbiotic function. Their number equals the total amount of cells of our own body, and they create an internal ecosystem in addition to the surrounding world.

Although high-throughput techniques, as an essential new step, are now broadly available to investigate the association of intestinal microorganisms with physiological phenomena or disease, the topic remains extremely complex regarding sample collection, analytical procedure, and evaluation of their functions as well as their integration and numerous interactions with human organs. Also, many bacterial metabolites ^152^, including dangerous ones, such as ammonia or hydrogen sulfide, and beneficial ones, such as short-chain fatty acids, must be considered. Last not least, T cells primed in the intestine can induce liver damage by aberrant homing in the liver ^153^. All of these different phenomena increase complexity. On the other hand, there is first evidence that common small molecules like N-acylamide with a signaling effect on G-protein-coupled receptors regulating metabolism are produced by human microbiota ^154^. Thus, in the end, a limited number of interacting biological structures may remain crucial.

It has been shown that alcohol intake affects the integrity of the gut from an early stage, on the one hand by altering the intestinal microbiota, including the small bowel and the oral cavity ^155, 156^—often coined dysbiosis—and on the other hand by increasing intestinal permeability ^157, 158^. As yet, it remains unclear which is the “chicken” and which is the “egg”, but both phenomena give rise to an increased transfer of inflammatory stimuli to the liver, mediated via Toll-like receptors, and to alteration of the bile acid pool and its enterohepatic circulation (for further reading, see 151, 159, 160). Moreover, fungal dysbiosis has been incriminated in the induction of liver damage in alcohol abuse ^161^.

A change in the individual bile acids by bacterial enzymes may lead to hepatobiliary injury via the induction of nuclear and G-protein-coupled cell surface receptors (see 162, 163). Based on these insights, numerous approaches, recently reviewed in depth ^159, 164– 166^, have been proposed to modulate the course of liver cirrhosis and portal hypertension.

Since a distinct change of the microbiota with a reduction of autochthonous bacteria has been found in patients with liver cirrhosis compared to healthy individuals ^167, 168^, ongoing trials are targeting intestinal dysbiosis in patients with liver cirrhosis via antibiotics, probiotics, or synbiotics (see 164). Most of these trials study surrogate markers. Results on hard clinical endpoints, such as liver failure, bleeding, or death, are still far from being reached. One promising randomized study ^169^ showed that the probiotic VSL#3 reduced liver disease severity and hospitalization in patients with mainly alcoholic liver cirrhosis in India. Another study from India found that administration of this probiotic increased the response rate to propranolol with respect to a decrease in HVPG ^170^.

In patients with liver cirrhosis and variceal bleeding ^171^ and in patients with decompensated liver cirrhosis ^172, 173^, systemic application of antibiotics, which affect other organs as well as the gut, increased survival. But direct studies on portal hemodynamics are sparse. Norfloxacin partially reversed the hyperdynamic state, while RAAS activation and portal hypertension were not influenced or only to a minor degree ^174^. Rifaximin is a non-absorbable antibiotic with proven effect on hepatic encephalopathy ^175^. While its effect in the intestine has not been fully elucidated, it has been found to reduce the production and absorption of gut-derived toxins and inflammatory stimuli, such as ammonia and endotoxin (see 176). Overall, its effects in the intestine may be more eubiotic than antibiotic ^177^. According to uncontrolled trials, rifaximin reduced plasma endotoxin levels and HVPG in alcohol-related decompensated liver cirrhosis ^178^. Furthermore, it lowered the 5-year cumulative probability of decompensation of cirrhosis, including bleeding and encephalopathy, and resulted in better survival ^179^. However, although the data are promising, they are from only one center and as yet remain uncontrolled. A randomized double-blind placebo-controlled trial consisting of a 4-week treatment with rifaximin found no effect on bacterial translocation, HVPG, systemic hemodynamics, kidney function, or vasoactive hormones, including plasma renin ^180^, and a further study found that there was no short-term effect of rifaximin on systemic inflammatory markers or intestinal bacterial composition ^181^.

Alteration of the enterohepatic circulation of bile acids in liver and biliary disease ^182– 184^ has also become a topic of research in portal hypertension (see above). Major regulators of bile acid homeostasis, such as the farnesoid X receptor (FXR) or TGR5 ^185, 186^, are addressed by specific drugs. Two different research groups found that in animal models of cirrhosis, farnesoid receptor agonists reduced portal hypertension ^187, 188^. However, there is still a long way to go from proof of this concept in rats to an established treatment in the clinical situation. Bearing in mind that the FXR agonist obeticholic acid is already used in phase III trials ^189^ for primary biliary cholangitis, particular emphasis should be given to determining to what extent these drugs may blunt portal hypertension and its complications. Further non-bile acid and non-steroidal FXR agonists with or without concomitant TGR5 activity are being tested in animal models ^188, 190^.

It has been shown in animals, and now even in a small series of humans, that transplant of fecal microbiota may reverse hepatic disease or its symptoms ^191^. Furthermore, transplantation of stool from eubiotic rats to animals with a NASH model of portal hypertension significantly reduced portal pressure ^144^. However, to date, it is difficult to imagine that such an approach will have a future in clinical routine.

Dysbiosis of the gut and alteration of intestinal permeability, which are associated with alcohol intake, affect not only the liver but also adipose tissue and the brain, with involvement of the autonomic nervous system as a regulating circuit, at least according to studies in animal models ^192– 196^. Activation of the immune system by the gut, including Toll-like receptor signaling, with release of proinflammatory cytokines is now regarded as a broad phenomenon that not only causes organ damage but also induces dysregulation in the brain, resulting in an unopposed craving for an unhealthy diet and liquids (see 197– 199). In this vicious cycle, portal hypertension presents as a late link to disease. Thus, it is eminently worthwhile to support beneficial change in the eating or drinking habits of patients, which are controlled by the brain. It has been shown that in patients with liver cirrhosis, weight loss ^200^ or abstinence from alcohol ^94, 201^ significantly reduces portal hypertension. This appears to be the most important strategy to influence the gut–liver axis and portal hypertension ( Figure 1).

## Other potential options and drugs

Patients with liver cirrhosis have an increased risk of portal vein thrombosis. The prevalence ranges from 10 to 20%, and the yearly incidence is estimated to be somewhat less than 10% ^202^. Anticoagulants reduce significantly the risk of occurrence ^203^ and recurrence ^204^. One small randomized study found not only a significantly reduced occurrence of portal vein thrombosis but also fewer events of decompensation and even improved survival in patients with advanced cirrhosis receiving low-molecular-weight heparin over a period of nearly one year ^203^. The authors explain the positive effect by better microcirculation of the gut and less translocation of bacteria. Moreover, enoxaparin reduced intrahepatic vascular resistance and fibrosis in animal models of liver cirrhosis ^205^. Further studies on the putative beneficial effect of anticoagulation in patients with liver cirrhosis are needed.

A recent comprehensive publication reviewed available studies on several other drugs that reduce portal hypertension, mostly in animal models ^206^ and still in an experimental stage. In fact, many of these drugs reduce intrahepatic resistance. However, care has to be taken to prevent these drugs from aggravating hyperdynamic cardiovascular effects outside the liver ^207– 209^ and from eliciting toxic effects inside the liver ^210– 214^. We have addressed this problem for many years ^122, 215– 223^. Unfortunately, clinical trials in humans are lacking. A search for the one panacea seems futile. The actual stage of portal hypertension and its etiology have to be considered. Interruption or suppression of viremia as a cause of chronic liver disease and portal hypertension is now possible for the hepatitis B as well as the hepatitis C virus. Thus, advanced liver disease and portal hypertension increasingly appear to be food-induced disorders rather than chronic infectious diseases. Here, the modulation of behavior is key, as it is the case with so many of the diseases of modern civilization. Drugs and interventions, however, retain their importance in treating the decompensated stage no matter whether initial etiology could be stopped or not (see above).

## Combined and stage-dependent treatment

At the stage of compensated cirrhosis without clinical signs of disease, it is crucial to halt progression. This is mainly achieved by interruption of an etiology that perpetuates inflammation and fibrogenesis leading to portal hypertension. Convincing examples include interruption or suppression of viremia, abstinence from alcohol abuse, immunosuppression of autoimmune liver disease, use of ursodeoxycholic acid in primary biliary cholangitis, or venesection for hemochromatosis. In all of these different etiologies, early diagnosis is important. However, co-factors (e.g. obesity, alcohol intake, or hepatotoxic drugs) which aggravate hepatic damage have to be considered and treated.

Once portal hypertension has developed, as documented by the invasive or non-invasive methods outlined above, it is desirable to blunt its interaction with systemic hemodynamics and to prevent further organ dysfunction. This applies not only to the liver but also to the heart, kidneys, brain, and lungs. At this stage, reduction of portal hypertension by modulation of the intestine as a source of inflammatory stimuli or retarding inflammatory pathways may be promising strategies. Here, statins, NSBB, FXR agonists, probiotics, and antibiotics have been established or appear to be promising.

In patients with ascites and variceal hemorrhage, early placement of a small lumen TIPS is an option because of its paramount effect on the prevention of bleeding and improvement of kidney function. However, once the stage of disease has become more advanced with threatening progress towards ACLF, survival is low and difficult to improve without liver transplantation. Thus, more effort must be put into early detection and prevention of liver disease, and more effective measures besides transplantation must be developed to treat decompensated cirrhosis. Combining small lumen TIPS with modulation of the systemic inflammatory response could be a possible approach.

## Conclusion

Portal hypertension is a surrogate of advanced liver disease. Reduction of portal pressure is the most efficient step to prevent intestinal bleeding and treat ascites. But this has a limited impact on survival. Interruption or modulation of inflammatory stimuli leading to liver damage and dysfunction of other organs is key in order to prevent death or liver transplantation as ultimate rescue.

## Abbreviations

ACLF, acute-on-chronic liver failure; ARFI, acoustic radiation force impulse imaging; FXR, farnesoid X receptor; HCC, hepatocellular carcinoma; HVPG, hepatic venous pressure gradient; MELD, model of end-stage liver disease; MR, magnetic resonance; MRI, magnetic resonance imaging; NSBB, non-selective beta-blockers; RAAS, renin–angiotensin–aldosterone system; SWE, shear wave elastography; TE, transient elastography; TIPS, transjugular intrahepatic portosystemic shunt.

## References

[1] Lebrec D Nouel O Corbic M: Propranolol--a medical treatment for portal hypertension? *Lancet.* 1980;2(8187):180–2. 10.1016/S0140-6736(80)90063-X 6105342

[2] Møller S Bendtsen F: The pathophysiology of arterial vasodilatation and hyperdynamic circulation in cirrhosis. *Liver Int.* 2018;38(4):570–80. 10.1111/liv.13589 28921803

[3] Wong F Blendis L: New challenge of hepatorenal syndrome: prevention and treatment. *Hepatology.* 2001;34(6):1242–51. 10.1053/jhep.2001.29200 11732014

[4] Moller S Henriksen JH: Cardiopulmonary complications in chronic liver disease. *World J Gastroenterol.* 2006;12(4):526–38. 10.3748/wjg.v12.i4.526 16489664 PMC4066083

[5] Fallon MB Abrams GA: Hepatopulmonary syndrome. *Curr Gastroenterol Rep.* 2000;2(1):40–5. 10981002 10.1007/s11894-000-0050-8

[6] Macías-Rodríguez RU Duarte-Rojo A Cantú-Brito C: Cerebral haemodynamics in cirrhotic patients with hepatic encephalopathy. *Liver Int.* 2015;35(2):344–52. 10.1111/liv.12557 24690075

[7] Mehta G Mookerjee RP Sharma V: Systemic inflammation is associated with increased intrahepatic resistance and mortality in alcohol-related acute-on-chronic liver failure. *Liver Int.* 2015;35(3):724–34. 10.1111/liv.12559 24703488

[8] Child CG Turcotte JG: Surgery and portal hypertension. *Major Probl Clin Surg.* 1964;1:1–85. 4950264

[9] D'Amico G Garcia-Tsao G Pagliaro L: Natural history and prognostic indicators of survival in cirrhosis: a systematic review of 118 studies. *J Hepatol.* 2006;44(1):217–31. 10.1016/j.jhep.2005.10.013 16298014

[10] North Italian Endoscopic Club for the Study and Treatment of Esophageal Varices: Prediction of the first variceal hemorrhage in patients with cirrhosis of the liver and esophageal varices. A prospective multicenter study. *N Engl J Med.* 1988;319(15):983–9. 10.1056/NEJM198810133191505 3262200

[11] de Franchis R, Baveno VI Faculty: Expanding consensus in portal hypertension: Report of the Baveno VI Consensus Workshop: Stratifying risk and individualizing care for portal hypertension. *J Hepatol.* 2015;63(3):743–52. 10.1016/j.jhep.2015.05.022 26047908

[12] Bosch J Sauerbruch T: Esophageal varices: Stage-dependent treatment algorithm. *J Hepatol.* 2016;64(3):746–8. 10.1016/j.jhep.2015.11.039 26810377

[13] Berzigotti A Seijo S Arena U: Elastography, spleen size, and platelet count identify portal hypertension in patients with compensated cirrhosis. *Gastroenterology.* 2013;144(1):102–111.e1. 10.1053/j.gastro.2012.10.001 23058320

[14] Frulio N Trillaud H: Ultrasound elastography in liver. *Diagn Interv Imaging.* 2013;94(5):515–34. 10.1016/j.diii.2013.02.005 23623211

[15] Dietrich CF Bamber J Berzigotti A: EFSUMB Guidelines and Recommendations on the Clinical Use of Liver Ultrasound Elastography, Update 2017 (Long Version). *Ultraschall Med.* 2017;38(4):e16–e47. 10.1055/s-0043-103952 28407655

[16] Ferraioli G Filice C Castera L: WFUMB guidelines and recommendations for clinical use of ultrasound elastography: Part 3: liver. *Ultrasound Med Biol.* 2015;41(5):1161–79. 10.1016/j.ultrasmedbio.2015.03.007 25800942

[17] European Association for Study of Liver, Asociacion Latinoamericana para el Estudio del Higado: EASL-ALEH Clinical Practice Guidelines: Non-invasive tests for evaluation of liver disease severity and prognosis. *J Hepatol.* 2015;63(1):237–64. 10.1016/j.jhep.2015.04.006 25911335

[18] Castera L: Non-invasive tests for liver fibrosis progression and regression. *J Hepatol.* 2016;64(1):232–3. 10.1016/j.jhep.2015.10.011 26603523

[19] Herrmann E de Lédinghen V Cassinotto C: Assessment of biopsy-proven liver fibrosis by two-dimensional shear wave elastography: An individual patient data-based meta-analysis. *Hepatology.* 2018;67(1):260–72. 10.1002/hep.29179 28370257 PMC5765493

[20] Kjærgaard M Thiele M Jansen C: High risk of misinterpreting liver and spleen stiffness using 2D shear-wave and transient elastography after a moderate or high calorie meal. *PLoS One.* 2017;12(4):e0173992. 10.1371/journal.pone.0173992 28376114 PMC5380309

[21] Jansen C Bogs C Verlinden W: Algorithm to rule out clinically significant portal hypertension combining Shear-wave elastography of liver and spleen: a prospective multicentre study. *Gut.* 2016;65(6):1057–8. 10.1136/gutjnl-2016-311536 26896458

[22] Jansen C Bogs C Verlinden W: Shear-wave elastography of the liver and spleen identifies clinically significant portal hypertension: A prospective multicentre study. *Liver Int.* 2017;37(3):396–405. 10.1111/liv.13243 27569696

[23] Kennedy P Wagner M Castéra L: Quantitative Elastography Methods in Liver Disease: Current Evidence and Future Directions. *Radiology.* 2018;286(3):738–63. 10.1148/radiol.2018170601 29461949 PMC5831316

[24] Block W Reichel C Träber F: Effect of cytochrome P450 induction on phosphorus metabolites and proton relaxation times measured by *in vivo* ^31^P-magnetic resonance spectroscopy and ^1^H-magnetic resonance relaxometry in human liver. *Hepatology.* 1997;26(6):1587–91. 10.1002/hep.510260629 9398002

[25] Reichel C Block W Skodra T: Relationship between cytochrome P-450 induction by rifampicin, hepatic volume and portal blood flow in man. *Eur J Gastroenterol Hepatol.* 1997;9(10):975–9. 9391787 10.1097/00042737-199710000-00010

[26] Petitclerc L Sebastiani G Gilbert G: Liver fibrosis: Review of current imaging and MRI quantification techniques. *J Magn Reson Imaging.* 2017;45(5):1276–95. 10.1002/jmri.25550 27981751

[27] Kukuk GM Hittatiya K Sprinkart AM: Comparison between modified Dixon MRI techniques, MR spectroscopic relaxometry, and different histologic quantification methods in the assessment of hepatic steatosis. *Eur Radiol.* 2015;25(10):2869–79. 10.1007/s00330-015-3703-6 25903702

[28] Luetkens JA Klein S Traeber F: Quantitative liver MRI including extracellular volume fraction for non-invasive quantification of liver fibrosis: a prospective proof-of-concept study. *Gut.* 2018;67(3):593–4. 10.1136/gutjnl-2017-314561 28754777

[29] Praktiknjo M Book M Luetkens J: Fat-free muscle mass in magnetic resonance imaging predicts acute-on-chronic liver failure and survival in decompensated cirrhosis. *Hepatology.* 2018;67(3):1014–26. 10.1002/hep.29602 29059469

[30] Groszmann RJ Wongcharatrawee S: The hepatic venous pressure gradient: anything worth doing should be done right. *Hepatology.* 2004;39(2):280–2. 10.1002/hep.20062 14767976

[31] Myers JD Taylor WJ: Occlusive hepatic venous catheterization in the study of the normal liver, cirrhosis of the liver and noncirrhotic portal hypertension. *Circulation.* 1956;13(3):368–80. 10.1161/01.CIR.13.3.368 13356395

[32] Groszmann RJ Glickman M Blei AT: Wedged and free hepatic venous pressure measured with a balloon catheter. *Gastroenterology.* 1979;76(2):253–8. 759258

[33] Boyer TD Triger DR Horisawa M: Direct transhepatic measurement of portal vein pressure using a thin needle. Comparison with wedged hepatic vein pressure. *Gastroenterology.* 1977;72(4 Pt 1):584–9. 10.1016/S0016-5085(77)80136-4 838210

[34] Brensing KA Neubrand M Textor J: Endoscopic manometry of esophageal varices: evaluation of a balloon technique compared with direct portal pressure measurement. *J Hepatol.* 1998;29(1):94–102. 10.1016/S0168-8278(98)80183-9 9696497

[35] Bosch J Abraldes JG Berzigotti A: The clinical use of HVPG measurements in chronic liver disease. *Nat Rev Gastroenterol Hepatol.* 2009;6(10):573–82. 10.1038/nrgastro.2009.149 19724251

[36] Sauerbruch T Mengel M Dollinger M: Prevention of Rebleeding From Esophageal Varices in Patients With Cirrhosis Receiving Small-Diameter Stents Versus Hemodynamically Controlled Medical Therapy. *Gastroenterology.* 2015;149(3):660–8.e1. 10.1053/j.gastro.2015.05.011 25989386

[37] Groszmann RJ Garcia-Tsao G Bosch J: Beta-blockers to prevent gastroesophageal varices in patients with cirrhosis. *N Engl J Med.* 2005;353(21):2254–61. 10.1056/NEJMoa044456 16306522

[38] Boyer TD: Changing clinical practice with measurements of portal pressure. *Hepatology.* 2004;39(2):283–5. 10.1002/hep.20037 14767977

[39] You MW Kim KW Pyo J: A Meta-analysis for the Diagnostic Performance of Transient Elastography for Clinically Significant Portal Hypertension. *Ultrasound Med Biol.* 2017;43(1):59–68. 10.1016/j.ultrasmedbio.2016.07.025 27751595

[40] Kim G Kim MY Baik SK: Transient elastography versus hepatic venous pressure gradient for diagnosing portal hypertension: a systematic review and meta-analysis. *Clin Mol Hepatol.* 2017;23(1):34–41. 10.3350/cmh.2016.0059 28263953 PMC5381827

[41] Pugh RN Murray-Lyon IM Dawson JL: Transection of the oesophagus for bleeding oesophageal varices. *Br J Surg.* 1973;60(8):646–9. 10.1002/bjs.1800600817 4541913

[42] Kamath PS Wiesner RH Malinchoc M: A model to predict survival in patients with end-stage liver disease. *Hepatology.* 2001;33(2):464–70. 10.1053/jhep.2001.22172 11172350

[43] Malinchoc M Kamath PS Gordon FD: A model to predict poor survival in patients undergoing transjugular intrahepatic portosystemic shunts. *Hepatology.* 2000;31(4):864–71. 10.1053/he.2000.5852 10733541

[44] Schepke M Roth F Fimmers R: Comparison of MELD, Child-Pugh, and Emory model for the prediction of survival in patients undergoing transjugular intrahepatic portosystemic shunting. *Am J Gastroenterol.* 2003;98(5):1167–74. 10.1111/j.1572-0241.2003.07515.x 12809844

[45] Rudler M Bureau C Carbonell N: Recalibrated MELD and hepatic encephalopathy are prognostic factors in cirrhotic patients with acute variceal bleeding. *Liver Int.* 2018;38(3):469–76. 10.1111/liv.13632 29164762

[46] Sharma P Schaubel DE Goodrich NP: Serum sodium and survival benefit of liver transplantation. *Liver Transpl.* 2015;21(3):308–13. 10.1002/lt.24063 25504743 PMC4354811

[47] van Vugt JLA Alferink LJM Buettner S: A model including sarcopenia surpasses the MELD score in predicting waiting list mortality in cirrhotic liver transplant candidates: a competing risk analysis in a national cohort. *J Hepatol.* 2018;68(4):707–714, pii: S0168-8278(17)32474-1. 10.1016/j.jhep.2017.11.030 29221886

[48] Wong F Liu P Blendis L: Sodium homeostasis with chronic sodium loading in preascitic cirrhosis. *Gut.* 2001;49(6):847–51. 10.1136/gut.49.6.847 11709521 PMC1728551

[49] Wong F O'Leary JG Reddy KR: Acute Kidney Injury in Cirrhosis: Baseline Serum Creatinine Predicts Patient Outcomes. *Am J Gastroenterol.* 2017;112(7):1103–10. 10.1038/ajg.2017.122 28440305

[50] Clària J Stauber RE Coenraad MJ: Systemic inflammation in decompensated cirrhosis: Characterization and role in acute-on-chronic liver failure. *Hepatology.* 2016;64(4):1249–64. 10.1002/hep.28740 27483394

[51] Hernaez R Solà E Moreau R: Acute-on-chronic liver failure: an update. *Gut.* 2017;66(3):541–53. 10.1136/gutjnl-2016-312670 28053053 PMC5534763

[52] Moreau R Jalan R Gines P: Acute-on-chronic liver failure is a distinct syndrome that develops in patients with acute decompensation of cirrhosis. *Gastroenterology.* 2013;144(7):1426–37, 1437.e1–9. 10.1053/j.gastro.2013.02.042 23474284

[53] Jansen C Möller P Meyer C: Increase in liver stiffness after transjugular intrahepatic portosystemic shunt is associated with inflammation and predicts mortality. *Hepatology.* 2018;67(4):1472–84. 10.1002/hep.29612 29059466

[54] Alcaraz-Quiles J Titos E Casulleras M: Polymorphisms in the IL-1 gene cluster influence systemic inflammation in patients at risk for acute-on-chronic liver failure. *Hepatology.* 2017;65(1):202–16. 10.1002/hep.28896 27775822

[55] Appenrodt B Grünhage F Gentemann MG: Nucleotide-binding oligomerization domain containing 2 ( *NOD2*) variants are genetic risk factors for death and spontaneous bacterial peritonitis in liver cirrhosis. *Hepatology.* 2010;51(4):1327–33. 10.1002/hep.23440 20087966

[56] Lutz P Krämer B Kaczmarek DJ: A variant in the *nuclear dot protein 52kDa* gene increases the risk for spontaneous bacterial peritonitis in patients with alcoholic liver cirrhosis. *Dig Liver Dis.* 2016;48(1):62–8. 10.1016/j.dld.2015.09.011 26493630

[57] Nischalke HD Berger C Aldenhoff K: Toll-like receptor (TLR) 2 promoter and intron 2 polymorphisms are associated with increased risk for spontaneous bacterial peritonitis in liver cirrhosis. *J Hepatol.* 2011;55(5):1010–6. 10.1016/j.jhep.2011.02.022 21356257

[58] Trebicka J: Predisposing Factors in Acute-on-Chronic Liver Failure. *Semin Liver Dis.* 2016;36(2):167–73. 10.1055/s-0036-1583195 27172359

[59] D'Amico G Morabito A D'Amico M: Clinical states of cirrhosis and competing risks. *J Hepatol.* 2018;68(3):563–76. 10.1016/j.jhep.2017.10.020 29111320

[60] Rastogi A Maiwall R Bihari C: Cirrhosis histology and Laennec staging system correlate with high portal pressure. *Histopathology.* 2013;62(5):731–41. 10.1111/his.12070 23470026

[61] Suk KT Kim DJ: Staging of liver fibrosis or cirrhosis: The role of hepatic venous pressure gradient measurement. *World J Hepatol.* 2015;7(3):607–15. 10.4254/wjh.v7.i3.607 25848485 PMC4381184

[62] Monescillo A Martínez-Lagares F Ruiz-del-Arbol L: Influence of portal hypertension and its early decompression by TIPS placement on the outcome of variceal bleeding. *Hepatology.* 2004;40(4):793–801. 15382120 10.1002/hep.20386

[63] Moitinho E Escorsell A Bandi JC: Prognostic value of early measurements of portal pressure in acute variceal bleeding. *Gastroenterology.* 1999;117(3):626–31. 10.1016/S0016-5085(99)70455-5 10464138

[64] Garcia-Tsao G Friedman S Iredale J: Now there are many (stages) where before there was one: In search of a pathophysiological classification of cirrhosis. *Hepatology.* 2010;51(4):1445–9. 10.1002/hep.23478 20077563 PMC2882065

[65] CONN HO LINDENMUTH WW: PROPHYLACTIC PORTACAVAL ANASTOMOSIS IN CIRRHOTIC PATIENTS WITH ESOPHAGEAL VARICES: A PROGRESS REPORT OF A CONTINUING STUDY. *N Engl J Med.* 1965;272:1255–63. 10.1056/NEJM196506172722402 14290546

[66] Resnick RH Chalmers TC Ishihara AM: A controlled study of the prophylactic portacaval shunt. A final report. *Ann Intern Med.* 1969;70(4):675–88. 10.7326/0003-4819-70-4-675 4890511

[67] Franchis R de Dell’Era A: Variceal Hemorrhage.Springer Science & Business Media;2014;266 10.1007/978-1-4939-0002-2

[68] Rosemurgy AS Frohman HA Teta AF: Prosthetic H-graft portacaval shunts vs transjugular intrahepatic portasystemic stent shunts: 18-year follow-up of a randomized trial. *J Am Coll Surg.* 2012;214(4):445–53; discussion 453–5. 10.1016/j.jamcollsurg.2011.12.042 22463885

[69] Orloff MJ: Fifty-three years' experience with randomized clinical trials of emergency portacaval shunt for bleeding esophageal varices in Cirrhosis: 1958-2011. *JAMA Surg.* 2014;149(2):155–69. 10.1001/jamasurg.2013.4045 24402314

[70] Rössle M: TIPS: 25 years later. *J Hepatol.* 2013;59(5):1081–93. 10.1016/j.jhep.2013.06.014 23811307

[71] Khan S Tudur Smith C Williamson P: Portosystemic shunts versus endoscopic therapy for variceal rebleeding in patients with cirrhosis. *Cochrane Database Syst Rev.* 2006; (4): CD000553. 10.1002/14651858.CD000553.pub2 17054131 PMC7045742

[72] Holster IL Tjwa ET Moelker A: Reply. *Hepatology.* 2016;64(5):1817–8. 10.1002/hep.28664 27240057

[73] Holster IL Tjwa ET Moelker A: Covered transjugular intrahepatic portosystemic shunt versus endoscopic therapy + β-blocker for prevention of variceal rebleeding. *Hepatology.* 2016;63(2):581–9. 10.1002/hep.28318 26517576

[74] Luo X Wang Z Tsauo J: Advanced Cirrhosis Combined with Portal Vein Thrombosis: A Randomized Trial of TIPS versus Endoscopic Band Ligation Plus Propranolol for the Prevention of Recurrent Esophageal Variceal Bleeding. *Radiology.* 2015;276(1):286–93. 10.1148/radiol.15141252 25759969

[75] Henderson JM Boyer TD Kutner MH: Distal splenorenal shunt versus transjugular intrahepatic portal systematic shunt for variceal bleeding: a randomized trial. *Gastroenterology.* 2006;130(6):1643–51. 10.1053/j.gastro.2006.02.008 16697728

[76] Gupta AC Wang W Shah C: Added Value of Covered Stents in Transjugular Intrahepatic Portosystemic Shunt: A Large Single-Center Experience. *Cardiovasc Intervent Radiol.* 2017;40(11):1723–31. 10.1007/s00270-017-1694-1 28512687

[77] Qi X Tian Y Zhang W: Covered *versus* bare stents for transjugular intrahepatic portosystemic shunt: an updated meta-analysis of randomized controlled trials. *Therap Adv Gastroenterol.* 2017;10(1):32–41. 10.1177/1756283X16671286 28286557 PMC5330607

[78] Wang Q Lv Y Bai M: Eight millimetre covered TIPS does not compromise shunt function but reduces hepatic encephalopathy in preventing variceal rebleeding. *J Hepatol.* 2017;67(3):508–16. 10.1016/j.jhep.2017.05.006 28506905

[79] Qi X Tian Y Zhang W: Covered TIPS for secondary prophylaxis of variceal bleeding in liver cirrhosis: A systematic review and meta-analysis of randomized controlled trials. *Medicine (Baltimore).* 2016;95(50):e5680. 10.1097/MD.0000000000005680 27977618 PMC5268064

[80] García-Pagán JC Caca K Bureau C: Early use of TIPS in patients with cirrhosis and variceal bleeding. *N Engl J Med.* 2010;362(25):2370–9. 10.1056/NEJMoa0910102 20573925

[81] Garcia-Pagán JC Di Pascoli M Caca K: Use of early-TIPS for high-risk variceal bleeding: results of a post-RCT surveillance study. *J Hepatol.* 2013;58(1):45–50. 10.1016/j.jhep.2012.08.020 22940408

[82] Thabut D Pauwels A Carbonell N: Cirrhotic patients with portal hypertension-related bleeding and an indication for early-TIPS: a large multicentre audit with real-life results. *J Hepatol.* 2017;68(1):73–81. pii: S0168-8278(17)32281-X. 10.1016/j.jhep.2017.09.002 28918131

[83] Brensing KA Raab P Textor J: Prospective evaluation of a clinical score for 60-day mortality after transjugular intrahepatic portosystemic stent-shunt: Bonn TIPSS early mortality analysis. *Eur J Gastroenterol Hepatol.* 2002;14(7):723–31. 10.1097/00042737-200207000-00003 12169980

[84] Trebicka J: Emergency TIPS in a Child-Pugh B patient: When does the window of opportunity open and close? *J Hepatol.* 2017;66(2):442–50. 10.1016/j.jhep.2016.10.025 27984174

[85] Wong F Sniderman K Liu P: The mechanism of the initial natriuresis after transjugular intrahepatic portosystemic shunt. *Gastroenterology.* 1997;112(3):899–907. 10.1053/gast.1997.v112.pm9041252 9041252

[86] Busk TM Bendtsen F Poulsen JH: Transjugular intrahepatic portosystemic shunt: impact on systemic hemodynamics and renal and cardiac function in patients with cirrhosis. *Am J Physiol Gastrointest Liver Physiol.* 2018;314(2):G275–G286. 10.1152/ajpgi.00094.2017 29074483

[87] Brensing KA Textor J Perz J: Long term outcome after transjugular intrahepatic portosystemic stent-shunt in non-transplant cirrhotics with hepatorenal syndrome: a phase II study. *Gut.* 2000;47(2):288–95. 10.1136/gut.47.2.288 10896924 PMC1727992

[88] Sanyal AJ: Pros and cons of TIPS for refractory ascites. *J Hepatol.* 2005;43(6):924–5. 10.1016/j.jhep.2005.09.006 16246451

[89] Salerno F Cammà C Enea M: Transjugular intrahepatic portosystemic shunt for refractory ascites: a meta-analysis of individual patient data. *Gastroenterology.* 2007;133(3):825–34. 10.1053/j.gastro.2007.06.020 17678653

[90] Bureau C Thabut D Oberti F: Transjugular Intrahepatic Portosystemic Shunts With Covered Stents Increase Transplant-Free Survival of Patients With Cirrhosis and Recurrent Ascites. *Gastroenterology.* 2017;152(1):157–63. 10.1053/j.gastro.2016.09.016 27663604

[91] Lens S Alvarado-Tapias E Mariño Z: Effects of All-Oral Anti-Viral Therapy on HVPG and Systemic Hemodynamics in Patients With Hepatitis C Virus-Associated Cirrhosis. *Gastroenterology.* 2017;153(5):1273–1283.e1. 10.1053/j.gastro.2017.07.016 28734831

[92] Mandorfer M Kozbial K Freissmuth C: Interferon-free regimens for chronic hepatitis C overcome the effects of portal hypertension on virological responses. *Aliment Pharmacol Ther.* 2015;42(6):707–18. 10.1111/apt.13315 26179884

[93] Mandorfer M Kozbial K Schwabl P: Sustained virologic response to interferon-free therapies ameliorates HCV-induced portal hypertension. *J Hepatol.* 2016;65(4):692–9. 10.1016/j.jhep.2016.05.027 27242316

[94] Klein CP Kalk JF Müting D: [The effect of alcohol on portal vein hemodynamics in nutritional-toxic liver cirrhosis]. *Dtsch Med Wochenschr.* 1993;118(4):89–93. 10.1055/s-2008-1059304 8428561

[95] Vaillant GE: A 60-year follow-up of alcoholic men. *Addiction.* 2003;98(8):1043–51. 10.1046/j.1360-0443.2003.00422.x 12873238

[96] Vaillant GE: The Natural History of Alcoholism Revisited. Harvard University Press;2009;463 Reference Source

[97] Sinagra E Perricone G D'Amico M: Systematic review with meta-analysis: the haemodynamic effects of carvedilol compared with propranolol for portal hypertension in cirrhosis. *Aliment Pharmacol Ther.* 2014;39(6):557–68. 10.1111/apt.12634 24461301

[98] Mandorfer M Reiberger T: Beta blockers and cirrhosis, 2016. *Dig Liver Dis.* 2017;49(1):3–10. 10.1016/j.dld.2016.09.013 27717792

[99] Schepke M Kleber G Nürnberg D: Ligation versus propranolol for the primary prophylaxis of variceal bleeding in cirrhosis. *Hepatology.* 2004;40(1):65–72. 10.1002/hep.20284 15239087

[100] Gluud LL Krag A: Banding ligation versus beta-blockers for primary prevention in oesophageal varices in adults. *Cochrane Database Syst Rev.* 2012; (8): CD004544. 10.1002/14651858.CD004544.pub2 22895942 PMC11382336

[101] Feu F García-Pagán JC Bosch J: Relation between portal pressure response to pharmacotherapy and risk of recurrent variceal haemorrhage in patients with cirrhosis. *Lancet.* 1995;346(8982):1056–9. 10.1016/S0140-6736(95)91740-3 7564785

[102] D'Amico G Garcia-Pagan JC Luca A: Hepatic vein pressure gradient reduction and prevention of variceal bleeding in cirrhosis: a systematic review. *Gastroenterology.* 2006;131(5):1611–24. 10.1053/j.gastro.2006.09.013 17101332

[103] Villanueva C Graupera I Aracil C: A randomized trial to assess whether portal pressure guided therapy to prevent variceal rebleeding improves survival in cirrhosis. *Hepatology.* 2017;65(5):1693–707. 10.1002/hep.29056 28100019

[104] Sauerbruch T: Continuation of nonselective beta-blockers for patients with liver cirrhosis and hemodynamic nonresponse? *Hepatology.* 2017;66(4):1362–3. 10.1002/hep.29394 28734120

[105] Villanueva C Graupera I Alvarado E: Reply. *Hepatology.* 2017;66(4):1363–4. 10.1002/hep.29397 28741289

[106] Madsen BS Havelund T Krag A: Targeting the gut-liver axis in cirrhosis: antibiotics and non-selective β-blockers. *Adv Ther.* 2013;30(7):659–70. 10.1007/s12325-013-0044-1 23881723

[107] Mookerjee RP Pavesi M Thomsen KL: Treatment with non-selective beta blockers is associated with reduced severity of systemic inflammation and improved survival of patients with acute-on-chronic liver failure. *J Hepatol.* 2016;64(3):574–82. 10.1016/j.jhep.2015.10.018 26519600

[108] Sersté T Melot C Francoz C: Deleterious effects of beta-blockers on survival in patients with cirrhosis and refractory ascites. *Hepatology.* 2010;52(3):1017–22. 10.1002/hep.23775 20583214

[109] Mandorfer M Bota S Schwabl P: Nonselective β blockers increase risk for hepatorenal syndrome and death in patients with cirrhosis and spontaneous bacterial peritonitis. *Gastroenterology.* 2014;146(7):1680–90.e1. 10.1053/j.gastro.2014.03.005 24631577

[110] Reiberger T Mandorfer M: Beta adrenergic blockade and decompensated cirrhosis. *J Hepatol.* 2017;66(4):849–59. 10.1016/j.jhep.2016.11.001 27864004

[111] Reiberger T Ulbrich G Ferlitsch A: Carvedilol for primary prophylaxis of variceal bleeding in cirrhotic patients with haemodynamic non-response to propranolol. *Gut.* 2013;62(11):1634–41. 10.1136/gutjnl-2012-304038 23250049

[112] Bhardwaj A Kedarisetty CK Vashishtha C: Carvedilol delays the progression of small oesophageal varices in patients with cirrhosis: a randomised placebo-controlled trial. *Gut.* 2017;66(10):1838–43. 10.1136/gutjnl-2016-311735 27298379

[113] Sinha R Lockman KA Mallawaarachchi N: Carvedilol use is associated with improved survival in patients with liver cirrhosis and ascites. *J Hepatol.* 2017;67(1):40–6. 10.1016/j.jhep.2017.02.005 28213164

[114] Njei B McCarty TR Garcia-Tsao G: Beta-blockers in patients with cirrhosis and ascites: type of beta-blocker matters. *Gut.* 2016;65(8):1393–4. 10.1136/gutjnl-2016-312129 27207973

[115] Li T Ke W Sun P: Carvedilol for portal hypertension in cirrhosis: systematic review with meta-analysis. *BMJ Open.* 2016;6(5):e010902. 10.1136/bmjopen-2015-010902 27147389 PMC4861122

[116] Groszmann RJ Bosch J: Portal Hypertension in the 21st Century: The proceedings of a symposium sponsored by Axcan Pharma Inc. and NicOx S.A., held in Montrél, Canada, April 2–4, 2004.Springer Science & Business Media;2011;366 10.1007/978-94-007-1042-9

[117] Bosch J Groszmann RJ Shah VH: Evolution in the understanding of the pathophysiological basis of portal hypertension: How changes in paradigm are leading to successful new treatments. *J Hepatol.* 2015;62(1 Suppl):S121–30. 10.1016/j.jhep.2015.01.003 25920081 PMC4519833

[118] Bedi O Dhawan V Sharma PL: Pleiotropic effects of statins: new therapeutic targets in drug design. *Naunyn Schmiedebergs Arch Pharmacol.* 2016;389(7):695–712. 10.1007/s00210-016-1252-4 27146293

[119] Oesterle A Laufs U Liao JK: Pleiotropic Effects of Statins on the Cardiovascular System. *Circ Res.* 2017;120(1):229–43. 10.1161/CIRCRESAHA.116.308537 28057795 PMC5467317

[120] Schierwagen R Uschner FE Magdaleno F: Rationale for the use of statins in liver disease. *Am J Physiol Gastrointest Liver Physiol.* 2017;312(5):G407–G412. 10.1152/ajpgi.00441.2016 28280144

[121] Trebicka J Schierwagen R: Statins, Rho GTPases and KLF2: new mechanistic insight into liver fibrosis and portal hypertension. *Gut.* 2015;64(9):1349–50. 10.1136/gutjnl-2014-308800 25596180

[122] Trebicka J Hennenberg M Laleman W: Atorvastatin lowers portal pressure in cirrhotic rats by inhibition of RhoA/Rho-kinase and activation of endothelial nitric oxide synthase. *Hepatology.* 2007;46(1):242–53. 10.1002/hep.21673 17596891

[123] Rombouts K Kisanga E Hellemans K: Effect of HMG-CoA reductase inhibitors on proliferation and protein synthesis by rat hepatic stellate cells. *J Hepatol.* 2003;38(5):564–72. 10.1016/S0168-8278(03)00051-5 12713866

[124] Uschner FE Ranabhat G Choi SS: Statins activate the canonical hedgehog-signaling and aggravate non-cirrhotic portal hypertension, but inhibit the non-canonical hedgehog signaling and cirrhotic portal hypertension. *Sci Rep.* 2015;5:14573. 10.1038/srep14573 26412302 PMC4585958

[125] Marrone G Maeso-Díaz R García-Cardena G: KLF2 exerts antifibrotic and vasoprotective effects in cirrhotic rat livers: behind the molecular mechanisms of statins. *Gut.* 2015;64(9):1434–43. 10.1136/gutjnl-2014-308338 25500203

[126] Trebicka J Hennenberg M Odenthal M: Atorvastatin attenuates hepatic fibrosis in rats after bile duct ligation via decreased turnover of hepatic stellate cells. *J Hepatol.* 2010;53(4):702–12. 10.1016/j.jhep.2010.04.025 20633948

[127] Klein S Klösel J Schierwagen R: Atorvastatin inhibits proliferation and apoptosis, but induces senescence in hepatic myofibroblasts and thereby attenuates hepatic fibrosis in rats. *Lab Invest.* 2012;92(10):1440–50. 10.1038/labinvest.2012.106 22890553

[128] Zafra C Abraldes JG Turnes J: Simvastatin enhances hepatic nitric oxide production and decreases the hepatic vascular tone in patients with cirrhosis. *Gastroenterology.* 2004;126(3):749–55. 10.1053/j.gastro.2003.12.007 14988829

[129] Abraldes JG Albillos A Bañares R: Simvastatin lowers portal pressure in patients with cirrhosis and portal hypertension: a randomized controlled trial. *Gastroenterology.* 2009;136(5):1651–8. 10.1053/j.gastro.2009.01.043 19208350

[130] Abraldes JG Villanueva C Aracil C: Addition of Simvastatin to Standard Therapy for the Prevention of Variceal Rebleeding Does Not Reduce Rebleeding but Increases Survival in Patients With Cirrhosis. *Gastroenterology.* 2016;150(5):1160–1170.e3. 10.1053/j.gastro.2016.01.004 26774179

[131] Chang FM Wang YP Lang HC: Statins decrease the risk of decompensation in hepatitis B virus- and hepatitis C virus-related cirrhosis: A population-based study. *Hepatology.* 2017;66(3):896–907. 10.1002/hep.29172 28318053

[132] Huang YW Lee CL Yang SS: Statins Reduce the Risk of Cirrhosis and Its Decompensation in Chronic Hepatitis B Patients: A Nationwide Cohort Study. *Am J Gastroenterol.* 2016;111(7):976–85. 10.1038/ajg.2016.179 27166128

[133] Mohanty A Tate JP Garcia-Tsao G: Statins Are Associated With a Decreased Risk of Decompensation and Death in Veterans With Hepatitis C-Related Compensated Cirrhosis. *Gastroenterology.* 2016;150(2):430–40.e1. 10.1053/j.gastro.2015.10.007 26484707 PMC4727998

[134] Simon TG Bonilla H Yan P: Atorvastatin and fluvastatin are associated with dose-dependent reductions in cirrhosis and hepatocellular carcinoma, among patients with hepatitis C virus: Results from ERCHIVES. *Hepatology.* 2016;64(1):47–57. 10.1002/hep.28506 26891205 PMC4917438

[135] Lewis JH Mortensen ME Zweig S: Efficacy and safety of high-dose pravastatin in hypercholesterolemic patients with well-compensated chronic liver disease: Results of a prospective, randomized, double-blind, placebo-controlled, multicenter trial. *Hepatology.* 2007;46(5):1453–63. 10.1002/hep.21848 17668878

[136] Chalasani N Aljadhey H Kesterson J: Patients with elevated liver enzymes are not at higher risk for statin hepatotoxicity. *Gastroenterology.* 2004;126(5):1287–92. 10.1053/j.gastro.2004.02.015 15131789

[137] Rodríguez S Raurell I Torres-Arauz M: A Nitric Oxide-Donating Statin Decreases Portal Pressure with a Better Toxicity Profile than Conventional Statins in Cirrhotic Rats. *Sci Rep.* 2017;7: 40461. 10.1038/srep40461 28084470 PMC5233977

[138] McConnell M Iwakiri Y: Biology of portal hypertension. *Hepatol Int.* 2018;12(Suppl 1):11–23. 10.1007/s12072-017-9826-x 29075990 PMC7090883

[139] Pasarín M La Mura V Gracia-Sancho J: Sinusoidal endothelial dysfunction precedes inflammation and fibrosis in a model of NAFLD. *PLoS One.* 2012;7(4):e32785. 10.1371/journal.pone.0032785 22509248 PMC3317918

[140] Steib CJ Schewe J Gerbes AL: Infection as a Trigger for Portal Hypertension. *Dig Dis.* 2015;33(4):570–6. 10.1159/000375352 26159275

[141] Grønbaek H Sandahl TD Mortensen C: Soluble CD163, a marker of Kupffer cell activation, is related to portal hypertension in patients with liver cirrhosis. *Aliment Pharmacol Ther.* 2012;36(2):173–80. 10.1111/j.1365-2036.2012.05134.x 22591184

[142] Henao-Mejia J Elinav E Jin C: Inflammasome-mediated dysbiosis regulates progression of NAFLD and obesity. *Nature.* 2012;482(7384):179–85. 10.1038/nature10809 22297845 PMC3276682

[143] Guo J Loke J Zheng F: Functional linkage of cirrhosis-predictive single nucleotide polymorphisms of Toll-like receptor 4 to hepatic stellate cell responses. *Hepatology.* 2009;49(3):960–8. 10.1002/hep.22697 19085953 PMC2891538

[144] García-Lezana T Raurell I Bravo M: Restoration of a healthy intestinal microbiota normalizes portal hypertension in a rat model of nonalcoholic steatohepatitis. *Hepatology.* 2018;67(4):1485–98. 10.1002/hep.29646 29113028

[145] Harris R Harman DJ Card TR: Prevalence of clinically significant liver disease within the general population, as defined by non-invasive markers of liver fibrosis: a systematic review. *Lancet Gastroenterol Hepatol.* 2017;2(4):288–97. 10.1016/S2468-1253(16)30205-9 28404158

[146] Bellentani S Saccoccio G Costa G: Drinking habits as cofactors of risk for alcohol induced liver damage. The Dionysos Study Group. *Gut.* 1997;41(6):845–50. 10.1136/gut.41.6.845 9462221 PMC1891602

[147] Stickel F Datz C Hampe J: Pathophysiology and Management of Alcoholic Liver Disease: Update 2016. *Gut Liver.* 2017;11(2):173–88. 10.5009/gnl16477 28274107 PMC5347641

[148] Nanji AA Su GL Laposata M: Pathogenesis of alcoholic liver disease--recent advances. *Alcohol Clin Exp Res.* 2002;26(5):731–6. 10.1111/j.1530-0277.2002.tb02598.x 12045483

[149] Fukui H Brauner B Bode JC: Plasma endotoxin concentrations in patients with alcoholic and non-alcoholic liver disease: reevaluation with an improved chromogenic assay. *J Hepatol.* 1991;12(2):162–9. 10.1016/0168-8278(91)90933-3 2050995

[150] Mandrekar P Szabo G: Signalling pathways in alcohol-induced liver inflammation. *J Hepatol.* 2009;50(6):1258–66. 10.1016/j.jhep.2009.03.007 19398236 PMC3342816

[151] Tilg H Cani PD Mayer EA: Gut microbiome and liver diseases. *Gut.* 2016;65(12):2035–44. 10.1136/gutjnl-2016-312729 27802157

[152] Verbeke KA Boobis AR Chiodini A: Towards microbial fermentation metabolites as markers for health benefits of prebiotics. *Nutr Res Rev.* 2015;28(1):42–66. 10.1017/S0954422415000037 26156216 PMC4501371

[153] Trivedi PJ Adams DH: Mucosal immunity in liver autoimmunity: a comprehensive review. *J Autoimmun.* 2013;46:97–111. 10.1016/j.jaut.2013.06.013 23891169

[154] Cohen LJ Esterhazy D Kim S: Commensal bacteria make GPCR ligands that mimic human signalling molecules. *Nature.* 2017;549(7670):48–53. 10.1038/nature23874 28854168 PMC5777231

[155] Bajaj JS Betrapally NS Hylemon PB: Salivary microbiota reflects changes in gut microbiota in cirrhosis with hepatic encephalopathy. *Hepatology.* 2015;62(4):1260–71. 10.1002/hep.27819 25820757 PMC4587995

[156] Qin N Yang F Li A: Alterations of the human gut microbiome in liver cirrhosis. *Nature.* 2014;513(7516):59–64. 10.1038/nature13568 25079328

[157] Donnadieu-Rigole H Pansu N Mura T: Beneficial Effect of Alcohol Withdrawal on Gut Permeability and Microbial Translocation in Patients with Alcohol Use Disorder. *Alcohol Clin Exp Res.* 2018;42(1):32–40. 10.1111/acer.13527 29030980

[158] Parlesak A Schäfer C Schütz T: Increased intestinal permeability to macromolecules and endotoxemia in patients with chronic alcohol abuse in different stages of alcohol-induced liver disease. *J Hepatol.* 2000;32(5):742–7. 10.1016/S0168-8278(00)80242-1 10845660

[159] Bluemel S Williams B Knight R: Precision medicine in alcoholic and nonalcoholic fatty liver disease via modulating the gut microbiota. *Am J Physiol Gastrointest Liver Physiol.* 2016;311(6):G1018–G1036. 10.1152/ajpgi.00245.2016 27686615 PMC5206291

[160] Arab JP Martin-Mateos RM Shah VH: Gut-liver axis, cirrhosis and portal hypertension: the chicken and the egg. *Hepatol Int.* 2018;12(Suppl 1):24–33. 10.1007/s12072-017-9798-x 28550391 PMC6876989

[161] Yang AM Inamine T Hochrath K: Intestinal fungi contribute to development of alcoholic liver disease. *J Clin Invest.* 2017;127(7):2829–41. 10.1172/JCI90562 28530644 PMC5490775

[162] Beuers U Trauner M Jansen P: New paradigms in the treatment of hepatic cholestasis: from UDCA to FXR, PXR and beyond. *J Hepatol.* 2015;62(1 Suppl):S25–37. 10.1016/j.jhep.2015.02.023 25920087

[163] Fickert P Wagner M: Biliary bile acids in hepatobiliary injury - What is the link? *J Hepatol.* 2017;67(3):619–31. 10.1016/j.jhep.2017.04.026 28712691

[164] Wiest R Albillos A Trauner M: Targeting the gut-liver axis in liver disease. *J Hepatol.* 2017;67(5):1084–103. 10.1016/j.jhep.2017.05.007 28526488

[165] Woodhouse CA Patel VC Singanayagam A: Review article: the gut microbiome as a therapeutic target in the pathogenesis and treatment of chronic liver disease. *Aliment Pharmacol Ther.* 2018;47(2):192–202. 10.1111/apt.14397 29083037

[166] Fukui H: Gut Microbiome-based Therapeutics in Liver Cirrhosis: Basic Consideration for the Next Step. *J Clin Transl Hepatol.* 2017;5(3):249–60. 10.14218/JCTH.2017.00008 28936406 PMC5606971

[167] Chen Y Yang F Lu H: Characterization of fecal microbial communities in patients with liver cirrhosis. *Hepatology.* 2011;54(2):562–72. 10.1002/hep.24423 21574172

[168] Bajaj JS Heuman DM Hylemon PB: Altered profile of human gut microbiome is associated with cirrhosis and its complications. *J Hepatol.* 2014;60(5):940–7. 10.1016/j.jhep.2013.12.019 24374295 PMC3995845

[169] Dhiman RK Rana B Agrawal S: Probiotic VSL#3 reduces liver disease severity and hospitalization in patients with cirrhosis: a randomized, controlled trial. *Gastroenterology.* 2014;147(6):1327–37.e3. 10.1053/j.gastro.2014.08.031 25450083

[170] Gupta N Kumar A Sharma P: Effects of the adjunctive probiotic VSL#3 on portal haemodynamics in patients with cirrhosis and large varices: a randomized trial. *Liver Int.* 2013;33(8):1148–57. 10.1111/liv.12172 23601333

[171] Bernard B Grangé JD Khac EN: Antibiotic prophylaxis for the prevention of bacterial infections in cirrhotic patients with gastrointestinal bleeding: a meta-analysis. *Hepatology.* 1999;29(6):1655–61. 10.1002/hep.510290608 10347104

[172] Fernández J Navasa M Planas R: Primary prophylaxis of spontaneous bacterial peritonitis delays hepatorenal syndrome and improves survival in cirrhosis. *Gastroenterology.* 2007;133(3):818–24. 10.1053/j.gastro.2007.06.065 17854593

[173] Moreau R Elkrief L Bureau C: A randomized trial of 6-month norfloxacin therapy in patients with Child-Pugh class C cirrhosis. *J Hepatol.* 2017;66(1):S1 10.1016/S0168-8278(17)30264-7

[174] Rasaratnam B Kaye D Jennings G: The effect of selective intestinal decontamination on the hyperdynamic circulatory state in cirrhosis. A randomized trial. *Ann Intern Med.* 2003;139(3):186–93. 10.7326/0003-4819-139-3-200308050-00008 12899586

[175] Bass NM Mullen KD Sanyal A: Rifaximin treatment in hepatic encephalopathy. *N Engl J Med.* 2010;362(12):1071–81. 10.1056/NEJMoa0907893 20335583

[176] Bajaj JS: Review article: potential mechanisms of action of rifaximin in the management of hepatic encephalopathy and other complications of cirrhosis. *Aliment Pharmacol Ther.* 2016;43 Suppl 1:11–26. 10.1111/apt.13435 26618922

[177] Ponziani FR Zocco MA D'Aversa F: Eubiotic properties of rifaximin: Disruption of the traditional concepts in gut microbiota modulation. *World J Gastroenterol.* 2017;23(25):4491–9. 10.3748/wjg.v23.i25.4491 28740337 PMC5504364

[178] Vlachogiannakos J Saveriadis AS Viazis N: Intestinal decontamination improves liver haemodynamics in patients with alcohol-related decompensated cirrhosis. *Aliment Pharmacol Ther.* 2009;29(9):992–9. 10.1111/j.1365-2036.2009.03958.x 19210289

[179] Vlachogiannakos J Viazis N Vasianopoulou P: Long-term administration of rifaximin improves the prognosis of patients with decompensated alcoholic cirrhosis. *J Gastroenterol Hepatol.* 2013;28(3):450–5. 10.1111/jgh.12070 23216382

[180] Kimer N Pedersen JS Busk TM: Rifaximin has no effect on hemodynamics in decompensated cirrhosis: A randomized, double-blind, placebo-controlled trial. *Hepatology.* 2017;65(2):592–603. 10.1002/hep.28898 27775818

[181] Kimer N Pedersen JS Tavenier J: Rifaximin has minor effects on bacterial composition, inflammation, and bacterial translocation in cirrhosis: A randomized trial. *J Gastroenterol Hepatol.* 2018;33(1):307–14. 10.1111/jgh.13852 28671712

[182] Paumgartner G: Pharmacotherapy of cholestatic liver diseases. *J Dig Dis.* 2010;11(3):119–25. 10.1111/j.1751-2980.2010.00427.x 20579215

[183] Trauner M Fuchs CD Halilbasic E: New therapeutic concepts in bile acid transport and signaling for management of cholestasis. *Hepatology.* 2017;65(4):1393–404. 10.1002/hep.28991 27997980

[184] Gonzalez FJ Jiang C Patterson AD: An Intestinal Microbiota-Farnesoid X Receptor Axis Modulates Metabolic Disease. *Gastroenterology.* 2016;151(5):845–59. 10.1053/j.gastro.2016.08.057 27639801 PMC5159222

[185] Chávez-Talavera O Tailleux A Lefebvre P: Bile Acid Control of Metabolism and Inflammation in Obesity, Type 2 Diabetes, Dyslipidemia, and Nonalcoholic Fatty Liver Disease. *Gastroenterology.* 2017;152(7):1679–1694.e3. 10.1053/j.gastro.2017.01.055 28214524

[186] Schubert K Olde Damink SWM von Bergen M: Interactions between bile salts, gut microbiota, and hepatic innate immunity. *Immunol Rev.* 2017;279(1):23–35. 10.1111/imr.12579 28856736

[187] Verbeke L Farre R Trebicka J: Obeticholic acid, a farnesoid X receptor agonist, improves portal hypertension by two distinct pathways in cirrhotic rats. *Hepatology.* 2014;59(6):2286–98. 10.1002/hep.26939 24259407

[188] Schwabl P Hambruch E Seeland BA: The FXR agonist PX20606 ameliorates portal hypertension by targeting vascular remodelling and sinusoidal dysfunction. *J Hepato.* 2017;66(4):724–33. 10.1016/j.jhep.2016.12.005 27993716

[189] Nevens F Andreone P Mazzella G: A Placebo-Controlled Trial of Obeticholic Acid in Primary Biliary Cholangitis. *N Engl J Med.* 2016;375(7):631–43. 10.1056/NEJMoa1509840 27532829

[190] Carino A Cipriani S Marchianò S: BAR502, a dual FXR and GPBAR1 agonist, promotes browning of white adipose tissue and reverses liver steatosis and fibrosis. *Sci Rep.* 2017;7: 42801. 10.1038/srep42801 28202906 PMC5311892

[191] Bajaj JS Kassam Z Fagan A: Fecal microbiota transplant from a rational stool donor improves hepatic encephalopathy: A randomized clinical trial. *Hepatology.* 2017;66(6):1727–38. 10.1002/hep.29306 28586116 PMC6102730

[192] Kang DJ Betrapally NS Ghosh SA: Gut microbiota drive the development of neuroinflammatory response in cirrhosis in mice. *Hepatology.* 2016;64(4):1232–48. 10.1002/hep.28696 27339732 PMC5033692

[193] Milanski M Arruda AP Coope A: Inhibition of hypothalamic inflammation reverses diet-induced insulin resistance in the liver. *Diabetes.* 2012;61(6):1455–62. 10.2337/db11-0390 22522614 PMC3357298

[194] Waise TMZ Toshinai K Naznin F: One-day high-fat diet induces inflammation in the nodose ganglion and hypothalamus of mice. *Biochem Biophys Res Commun.* 2015;464(4):1157–62. 10.1016/j.bbrc.2015.07.097 26208455

[195] Thaler JP Yi C Schur EA: Obesity is associated with hypothalamic injury in rodents and humans. *J Clin Invest.* 2012;122(1):153–62. 10.1172/JCI59660 22201683 PMC3248304

[196] Gardemann A Püschel GP Jungermann K: Nervous control of liver metabolism and hemodynamics. *Eur J Biochem.* 1992;207(2):399–411. 10.1111/j.1432-1033.1992.tb17063.x 1633798

[197] Leclercq S de Timary P Delzenne NM: The link between inflammation, bugs, the intestine and the brain in alcohol dependence. *Transl Psychiatry.* 2017;7(2):e1048. 10.1038/tp.2017.15 28244981 PMC5545644

[198] Temko JE Bouhlal S Farokhnia M: The Microbiota, the Gut and the Brain in Eating and Alcohol Use Disorders: A 'Ménage à Trois'? *Alcohol Alcohol.* 2017;52(4):403–13. 10.1093/alcalc/agx024 28482009 PMC5860274

[199] Velloso LA Folli F Saad MJ: TLR4 at the Crossroads of Nutrients, Gut Microbiota, and Metabolic Inflammation. *Endocr Rev.* 2015;36(3):245–71. 10.1210/er.2014-1100 25811237

[200] Berzigotti A Albillos A Villanueva C: Effects of an intensive lifestyle intervention program on portal hypertension in patients with cirrhosis and obesity: The SportDiet study. *Hepatology.* 2017;65(4):1293–305. 10.1002/hep.28992 27997989

[201] Müting D Kalk JF Fischer R: Spontaneous regression of oesophageal varices after long-term conservative treatment. Retrospective study in 20 patients with alcoholic liver cirrhosis, posthepatitic cirrhosis and haemochromatosis with cirrhosis. *J Hepatol.* 1990;10(2):158–62. 10.1016/0168-8278(90)90045-S 2332585

[202] Francoz C Valla D Durand F: Portal vein thrombosis, cirrhosis, and liver transplantation. *J Hepatol.* 2012;57(1):203–12. 10.1016/j.jhep.2011.12.034 22446690

[203] Villa E Cammà C Marietta M: Enoxaparin prevents portal vein thrombosis and liver decompensation in patients with advanced cirrhosis. *Gastroenterology.* 2012;143(5):1253–60.e1-4. 10.1053/j.gastro.2012.07.018 22819864

[204] Loffredo L Pastori D Farcomeni A: Effects of Anticoagulants in Patients With Cirrhosis and Portal Vein Thrombosis: A Systematic Review and Meta-analysis. *Gastroenterology.* 2017;153(2):480–487.e1. 10.1053/j.gastro.2017.04.042 28479379

[205] Cerini F Vilaseca M Lafoz E: Enoxaparin reduces hepatic vascular resistance and portal pressure in cirrhotic rats. *J Hepatol.* 2016;64(4):834–42. 10.1016/j.jhep.2015.12.003 26686269

[206] Schwabl P Laleman W: Novel treatment options for portal hypertension. *Gastroenterol Rep (Oxf).* 2017;5(2):90–103. 10.1093/gastro/gox011 28533907 PMC5421460

[207] Heller J Shiozawa T Trebicka J: Acute haemodynamic effects of losartan in anaesthetized cirrhotic rats. *Eur J Clin Invest.* 2003;33(11):1006–12. 10.1046/j.1365-2362.2003.01251.x 14636305

[208] Hennenberg M Biecker E Trebicka J: Defective RhoA/Rho-kinase signaling contributes to vascular hypocontractility and vasodilation in cirrhotic rats. *Gastroenterology.* 2006;130(3):838–54. 10.1053/j.gastro.2005.11.029 16530523

[209] Hennenberg M Trebicka J Kohistani Z: Hepatic and HSC-specific sorafenib effects in rats with established secondary biliary cirrhosis. *Lab Invest.* 2011;91(2):241–51. 10.1038/labinvest.2010.148 20921950

[210] Berzigotti A Bellot P De Gottardi A: NCX-1000, a nitric oxide-releasing derivative of UDCA, does not decrease portal pressure in patients with cirrhosis: results of a randomized, double-blind, dose-escalating study. *Am J Gastroenterol.* 2010;105(5):1094–101. 10.1038/ajg.2009.661 19920806

[211] Biecker E Trebicka J Kang A: Treatment of bile duct-ligated rats with the nitric oxide synthase transcription enhancer AVE 9488 ameliorates portal hypertension. *Liver Int.* 2008;28(3):331–8. 10.1111/j.1478-3231.2008.01664.x 18290775

[212] Fiorucci S Antonelli E Morelli O: NCX-1000, a NO-releasing derivative of ursodeoxycholic acid, selectively delivers NO to the liver and protects against development of portal hypertension. *Proc Natl Acad Sci U S A.* 2001;98(15):8897–902. 10.1073/pnas.151136298 11447266 PMC37532

[213] Fiorucci S Antonelli E Brancaleone V: NCX-1000, a nitric oxide-releasing derivative of ursodeoxycholic acid, ameliorates portal hypertension and lowers norepinephrine-induced intrahepatic resistance in the isolated and perfused rat liver. *J Hepatol.* 2003;39(6):932–9. 10.1016/S0168-8278(03)00393-3 14642608

[214] Zhou Q Hennenberg M Trebicka J: Intrahepatic upregulation of RhoA and Rho-kinase signalling contributes to increased hepatic vascular resistance in rats with secondary biliary cirrhosis. *Gut.* 2006;55(9):1296–305. 10.1136/gut.2005.081059 16492715 PMC1860046

[215] Grace JA Klein S Herath CB: Activation of the MAS receptor by angiotensin-(1-7) in the renin-angiotensin system mediates mesenteric vasodilatation in cirrhosis. *Gastroenterology.* 2013;145(4):874–884.e5. 10.1053/j.gastro.2013.06.036 23796456

[216] Granzow M Schierwagen R Klein S: Angiotensin-II type 1 receptor-mediated Janus kinase 2 activation induces liver fibrosis. *Hepatology.* 2014;60(1):334–48. 10.1002/hep.27117 24619965 PMC5512562

[217] Heller J Trebicka J Shiozawa T: Vascular, hemodynamic and renal effects of low-dose losartan in rats with secondary biliary cirrhosis. *Liver Int.* 2005;25(3):657–66. 10.1111/j.1478-3231.2005.01053.x 15910503

[218] Hennenberg M Trebicka J Stark C: Sorafenib targets dysregulated Rho kinase expression and portal hypertension in rats with secondary biliary cirrhosis. *Br J Pharmacol.* 2009;157(2):258–70. 10.1111/j.1476-5381.2009.00158.x 19338580 PMC2697813

[219] Klein S Herath CB Schierwagen R: Hemodynamic Effects of the Non-Peptidic Angiotensin-(1-7) Agonist AVE0991 in Liver Cirrhosis. *PLoS One.* 2015;10(9):e0138732. 10.1371/journal.pone.0138732 26406236 PMC4583473

[220] Klein S Rick J Lehmann J: Janus-kinase-2 relates directly to portal hypertension and to complications in rodent and human cirrhosis. *Gut.* 2017;66(1):145–55. 10.1136/gutjnl-2015-309600 26385087

[221] Kreisel W Deibert P Kupcinskas L: The phosphodiesterase-5-inhibitor udenafil lowers portal pressure in compensated preascitic liver cirrhosis. A dose-finding phase-II-study. *Dig Liver Dis.* 2015;47(2):144–50. 10.1016/j.dld.2014.10.018 25483910

[222] Trebicka J Leifeld L Hennenberg M: Hemodynamic effects of urotensin II and its specific receptor antagonist palosuran in cirrhotic rats. *Hepatology.* 2008;47(4):1264–76. 10.1002/hep.22170 18318439

[223] Trebicka J Hennenberg M Schulze Pröbsting A: Role of beta3-adrenoceptors for intrahepatic resistance and portal hypertension in liver cirrhosis. *Hepatology.* 2009;50(6):1924–35. 10.1002/hep.23222 19842096

